# Bacterial effector NleL promotes enterohemorrhagic *E. coli*-induced attaching and effacing lesions by ubiquitylating and inactivating JNK

**DOI:** 10.1371/journal.ppat.1006534

**Published:** 2017-07-28

**Authors:** Xiangpeng Sheng, Qing You, Hongnian Zhu, ZeNan Chang, Qingrun Li, Haifeng Wang, Chen Wang, Hongyan Wang, Lijian Hui, Chongtao Du, Xiaoduo Xie, Rong Zeng, Anning Lin, Dongfang Shi, Kangcheng Ruan, Jinghua Yan, George Fu Gao, Feng Shao, Ronggui Hu

**Affiliations:** 1 State Key Laboratory of Molecular Biology, CAS Center for Excellence in Molecular Cell Science, Innovation Center for Cell Signaling Network, Institute of Biochemistry and Cell Biology, Shanghai Institutes for Biological Sciences, Chinese Academy of Sciences, Shanghai, China; 2 University of Chinese Academy of Sciences, Beijing, China; 3 College of Chemistry, Chemical Engineering and Biotechnology, Donghua University, Shanghai, China; 4 David Geffen School of Medicine, University of California-Los Angeles, Los Angeles, California, United States of America; 5 State Key Laboratory of Cell Biology, Institute of Biochemistry and Cell Biology, Shanghai Institutes for Biological Sciences, Chinese Academy of Sciences, Shanghai, China; 6 College of Veterinary Medicine, Jilin University, Changchun, Jilin, China; 7 Ben May Department for Cancer Research, University of Chicago, Chicago, Illinois, United States of America; 8 College of Veterinary Medicine, Northeast Agricultural University, Harbin, Heilongjiang, China; 9 Beijing Institute of microbiology, Chinese Academy of Sciences, Beijing, China; 10 National Institute of Biological Sciences, Beijing, China; University of Utah, UNITED STATES

## Abstract

As a major diarrheagenic human pathogen, enterohemorrhagic *Escherichia coli* (EHEC) produce attaching and effacing (A/E) lesions, characterized by the formation of actin pedestals, on mammalian cells. A bacterial T3SS effector NleL from EHEC O157:H7 was recently shown to be a HECT-like E3 ligase *in vitro*, but its biological functions and host targets remain elusive. Here, we report that NleL is required to effectively promote EHEC-induced A/E lesions and bacterial infection. Furthermore, human c-Jun NH2-terminal kinases (JNKs) were identified as primary substrates of NleL. NleL-induced JNK ubiquitylation, particularly mono-ubiquitylation at the Lys 68 residue of JNK, impairs JNK’s interaction with an upstream kinase MKK7, thus disrupting JNK phosphorylation and activation. This subsequently suppresses the transcriptional activity of activator protein-1 (AP-1), which modulates the formation of the EHEC-induced actin pedestals. Moreover, JNK knockdown or inhibition in host cells complements NleL deficiency in EHEC infection. Thus, we demonstrate that the effector protein NleL enhances the ability of EHEC to infect host cells by targeting host JNK, and elucidate an inhibitory role of ubiquitylation in regulating JNK phosphorylation.

## Author summary

Enterohemorrhagic *Escherichia coli* (EHEC) can cause attaching and effacing (A/E) lesions to form in the colons of animals and humans, contributing to severe bacterial infection. NleL, an E3 ubiquitin ligase from EHEC O157:H7 is one of the bacterial type III secretion effectors that may be involved in the regulation of A/E lesions. However, NleL’s exact host targets and the detailed mechanistic actions are still unclear. Here, we report that the effector protein NleL effectively promotes EHEC-induced A/E lesions and bacterial infection by targeting the host JNK protein. Specifically, we find that NleL-mediated JNK ubiquitylation abolishes phosphorylation and activation of host JNK, subsequently suppressing the host JNK/AP-1 signaling pathway to favor the formation of EHEC-mediated actin pedestals on the surface of mammalian cells. Collectively, our work has not only discovered the A/E lesion-promoting function of NleL during EHEC infection, but also revealed a novel regulatory mechanism of host JNK protein.

## Introduction

**E**HEC is a globally spread, pathogenic *Escherichia coli* that infects animals and humans [1,2]. Particularly, O157:H7, as the most prominent serotype in the EHEC group, is a leading cause of diarrhea or hemorrhagic colitis in humans [3]. These pathogens belong to a distinct family of enteric bacteria that cause marked cytoskeletal changes and form unique attaching and effacing (A/E) lesions on intestinal epithelium [4,5]. A/E lesions are characterized by effacement of microvilli, intimate adherence between the bacterium and the host cell membrane, and the generation of actin pedestals, polymerized actin structures beneath the adherent bacteria [2]. Although the specific functions of actin pedestals are currently unclear [6], many studies suggest that the capability of A/E pathogens to form actin pedestals correlates with their ability to cause disease in hosts [7–9]. The type III protein secretion system (T3SS), as well as additional EHEC effector proteins, were reported to be involved in actin pedestal formation [10–12], but it remains incompletely understood how pedestal formation can be modulated.

**Fig 1:**
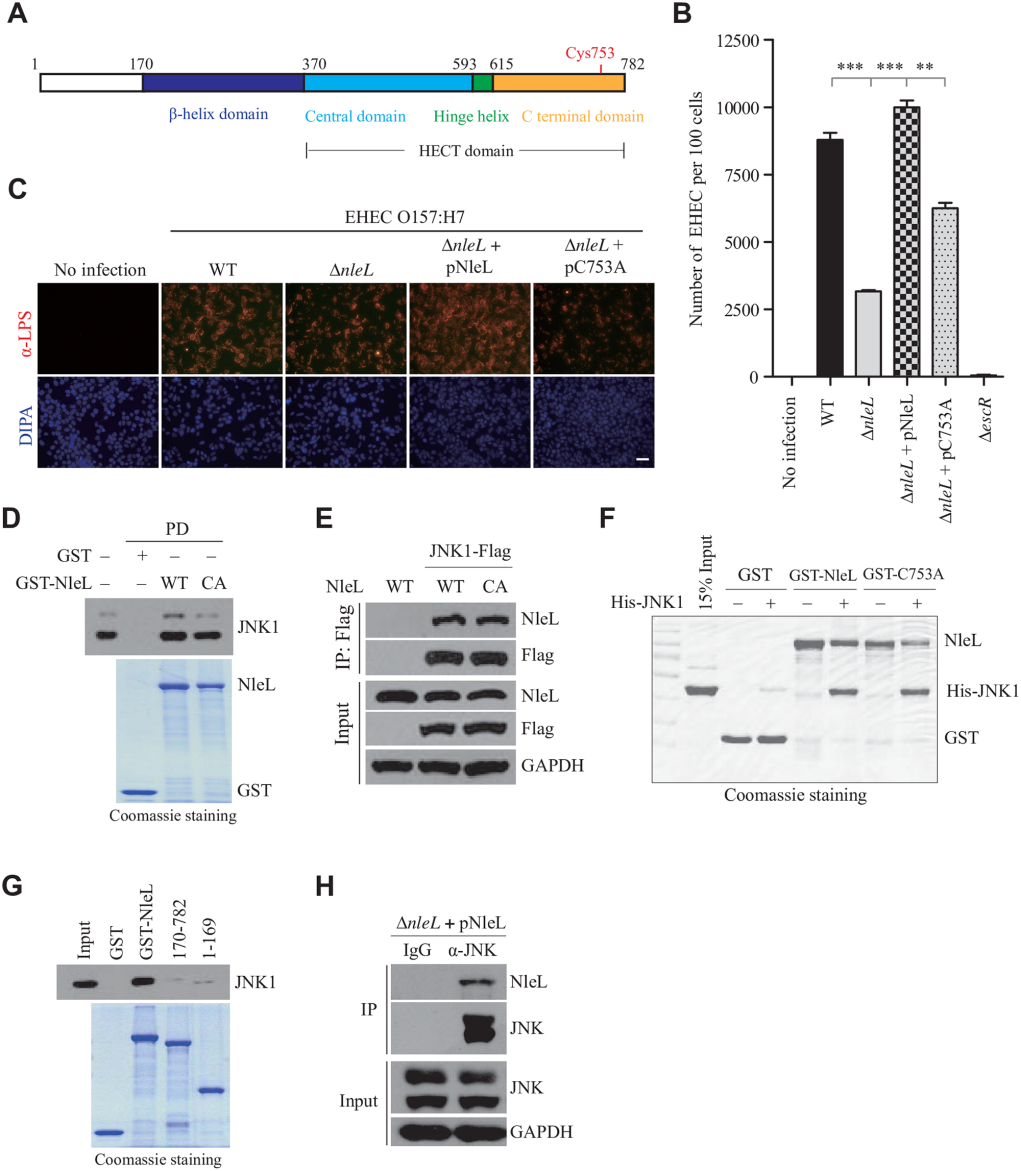
NleL contributes to EHEC attachment to host, and interacts with human JNK1 protein. **(A)** Domain structure of NleL from *E*. *coli* O157:H7. Cys753 in the C-terminal domain of NleL is the catalytic site responsible for the E3 Ub ligase activity of NleL. **(B)** Quantification of EHEC O157 attached to HeLa cells by colony formation assay. HeLa cells were infected with indicated EHEC strains with multiplicity of infection (MOI) of 100:1 for 2.5 h, washed with PBS and then re-cultured 2 h in fresh DMEM medium. Infected HeLa cells were thoroughly washed with PBS and aseptically lysed in 1% Triton X-100 buffer. Then bacterial colonies were determined after HeLa lysate being plated onto LB agar. Bars represent mean ± s.d. from three biological replicates, ***P < 0.001 (Student’s *t*-test, n = 3). **(C)** NleL promoted attachment of EHEC to mammalian cells. Infected HEK293T cells were thoroughly washed with PBS and subjected to immunofluorescence microscopy with anti-LPS antibody (in red). Scale bar, 50 μm. **(D)** Interaction of GST-NleL with endogenous JNK1. GST–tagged wild type or C753A mutant of NleL (GST-NleL-CA) were mixed with HEK293T cell lysates and the bound proteins were immunoblotted with the antibody specific for JNK1. GST, GST-tagged wild-type NleL or its C753A mutant were expressed and purified from bacteria. The C753A mutant was enzymatically dead and generated by substitution of the active site Cys753 with Ala. **(E)** NleL interacted with JNK1 *in vivo*. Flag-tagged JNK1, His_6_-tagged NleL (His-NleL) or its C753A mutant (His-NleL-CA) were ectopically expressed in HEK293T cells. Cells were lysed and subjected to immunoprecipitation (IP) with anti-Flag beads, followed by immunoblotting (IB) analysis with indicated antibodies. **(F)** Human JNK1 interacted with NleL *in vitro*. His_6_-tagged JNK1 (His-JNK1), GST-tagged wild-type NleL or its C753A mutant were purified from bacteria. **(G)** GST pull-down assay of full-length NleL or its truncations with JNK1. GST-tagged full-length NleL or its truncation mutants were individually mixed with lysates of the HEK293T cells expressing JNK1. After pull-down, Flag-tagged JNK1 was detected by IB analysis with anti-Flag. **(H)** Flag-tagged NleL secreted by EHEC O157 interacted with JNK in HEK293T cells. HEK293T cells were infected by EHEC strain expressing Flag-tagged NleL. Infected cells were thoroughly washed with PBS and lysed in IP buffer, and then IP with anti-JNK antibody and IB with anti-Flag antibody were successively performed. Data shown here are representative of at least three independent experiments.

Although the ubiquitin (Ub) system is exclusive to eukaryotes, prokaryotic bacteria have produced many E3 ligase-like effectors [13,14]. Recently, a bacterial T3SS effector NleL (Non-Lee-encoded effector ligase; also named EspX7) from EHEC O157:H7 was shown to be a HECT-like E3 ligase *in vitro*, with Cys753 as the catalytic site (Fig 1A) [15]. Later biochemical work revealed that NleL interacts with human E2 UbcH7 and is capable of assembling heterotypic Ub chains *in vitro* [16–18]. While NleL has been proposed to modulate EHEC-induced actin-pedestal formation [19], NleL’s specific host targets and functions in EHEC infection remain elusive.

In this study, we have identified human JNK as the first substrate of the bacterial E3 ligase NleL. The JNK (also known as stress-activated protein kinase, SAPK) family includes three highly homologous isoforms: ubiquitously expressed JNK1 and JNK2, and tissue-specific JNK3 [20]. JNKs are phosphorylated and activated by upstream kinases and regulate a wide range of cellular functions [21]. However, little is known about post-translational modifications other than phosphorylation regulating JNK functions. Here, we report that JNK proteins are ubiquitylated and inactivated by a bacterial effector NleL in EHEC infection, which promotes EHEC-induced A/E lesion formation and infection.

## Results

### NleL enhances adherence of EHEC O157:H7 to mammalian cells

To evaluate the effect of NleL on EHEC infection, a *nleL*-deletion mutant (Δ*nleL*) was first constructed by chromosomally inactivating the *nleL* gene in the parental EHEC O157:H7 Sakai strain (RIMD 0509952) as described previously [22]. Deletion of *nleL* has no effect on the growth of EHEC in culture medium (S1A Fig). We then assessed the ability of EHEC strains to infect mammalian cells. As a hallmark of EHEC infection, bacteria closely attach to cultured mammalian cells [23]. As shown, deletion of *nleL* from EHEC significantly reduced bacterial attachment to mammalian cells. More importantly, complementation of the *nleL*-deletion strain with wild-type NleL (Δ*nleL* + pNleL), but not the enzymatically-dead NleL mutant C753A (where the active site Cys at position 753 is replaced with Ala) (Δ*nleL* + pC753A), effectively restored the strong adherence of EHEC to host cells (Fig 1B and 1C). These data indicate that the bacterial effector NleL enhances the ability of EHEC to attach to mammalian cells in a manner dependent on its E3 ligase activity.

### NleL interacts with human JNK1 *in vitro* and *in vivo*

To identify the host targets of NleL, a human ORFs library was screened by a yeast two-hybrid (Y2H) system with full-length NleL as the bait. A cDNA encoding human JNK1, *Mapk8*, was identified in the Y2H screen (S1B Fig). A series of assays were then carried out to confirm the interaction of NleL with JNK1. Both wild-type NleL and the mutant C753A (also NleL-CA) readily co-immunoprecipitated with JNK1, suggesting NleL could form a complex with JNK1 independent of its E3 activity (Fig 1D and 1E and S1C Fig). A GST pull-down assay with the recombinant proteins confirmed that JNK1 can directly interact with NleL or its C753A mutant *in vitro* (Fig 1F). Compared to NleL_170–782_, a truncation mutant of NleL frequently used in structural or *in vitro* biochemical analyses [15,17], full-length NleL was shown to interact with JNK1 with significantly higher affinity (Fig 1G and S1D Fig), suggesting that the N-terminal unordered region of NleL might be involved in the interaction with JNK1. Moreover, we further demonstrated that endogenous JNKs interact with secreted NleL from EHEC in the infected mammalian cells (Fig 1H). Altogether, NleL interacts with host protein JNK1, providing a physical basis for their potential functional interplay.

### NleL ubiquitylates human JNK1

**Fig 2:**
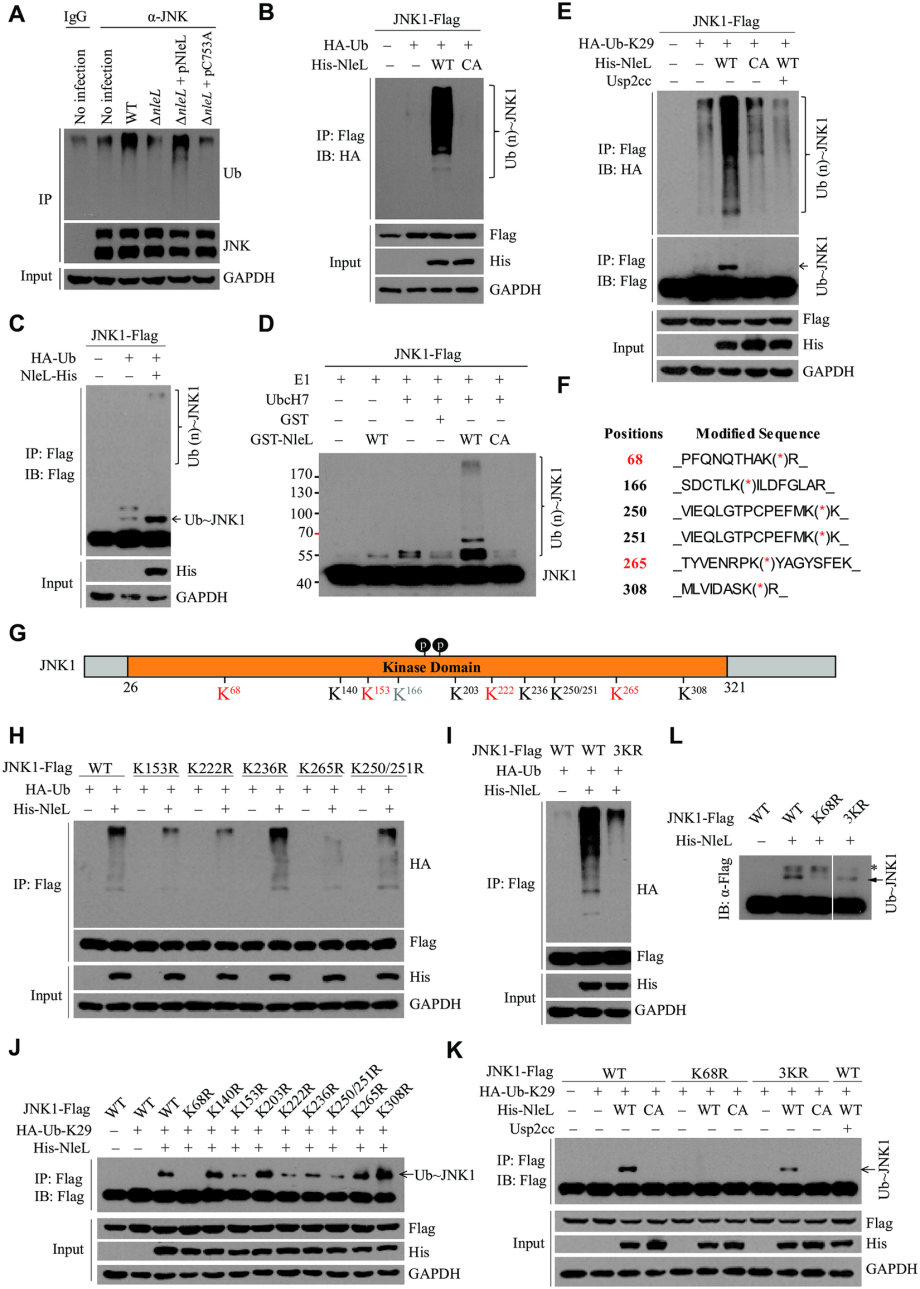
NleL ubiquitylates human JNK1. **(A)** NleL ubiquitylated endogenous JNK during EHEC O157:H7 infection. HEK293T cells were infected with EHEC strains with MOI of 100:1. 2.5 h after infection, cells were washed with PBS and cultured in the fresh DMEM medium for 2 h. Cells were lysed and subjected to IP with anti-JNK antibody and IB with anti-Ub antibody. **(B)** NleL ubiquitylated JNK1 *in vivo*. HEK293T cells were co-transfected with plasmids encoding HA-tagged Ub, Flag-tagged JNK1, His_6_-tagged wild-type NleL or its C753A mutant. Flag-tagged JNK1 was immunoprecipitated with anti-Flag M2 beads in denaturing RIPA buffer, followed by IB analyses with anti-HA antibody. **(C)** NleL induced mono- and poly-ubiquitylation of JNK1 *in vivo*. HEK293T cells were co-transfected with plasmids encoding HA-tagged Ub, Flag-tagged JNK1 and His_6_-tagged NleL. Flag-tagged JNK1 was immunoprecipitated with anti-Flag beads in denaturing RIPA buffer, followed by immunoblotting with anti-Flag antibody. **(D)** NleL ubiquitylated JNK1 *in vitro*. Flag-tagged JNK1 was expressed in HEK293T cells and purified with anti-Flag M2 affinity beads, and were subjected to *in vitro* ubiquitylation reaction mixture with indicated components. After 60 min incubation, the reactions were stopped and subjected to IB analysis with anti-Flag antibody. **(E)** NleL promoted mono- and poly-ubiquitylation of JNK1 with K29-Ub. HEK293T cells were co-transfected with plasmids encoding HA-tagged Lys 29 only Ub mutant (HA-K29-Ub), Flag-tagged JNK1, His_6_-tagged wild-type NleL or its C753A mutant. After immunoprecipitation, immunoblotting analyses with indicated antibodies were performed. Usp2cc, the catalytical core of human deubiquitylating enzyme USP2. **(F)** Mapping the sites for NleL-mediated ubiquitylation of JNK1 through mass spectrometry (MS) analyses. The immunoprecipitated JNK1 from HEK293T cells expressing NleL was subjected to MS analysis to determine the potential sites for NleL-mediated ubiquitylation. **(G)** A schematic view of the potential ubiquitylation sites of JNK1. The residues K68, K153, K222, and K265 were highlighted in red color to indicate the major ubiquitylation sites. **(H)** NleL promoted poly-Ub chains on JNK1 at multiple sites. HEK293T cells were transfected with His_6_-tagged NleL and Flag-tagged wild-type JNK1 or the mutants with Lys-to-Arg substitution at indicated sites. **(I)** NleL-mediated poly-ubiquitylation of JNK1 was almost abolished by simultaneous Lys-to-Arg substitutions at three major ubiquitylation sites (K153R/ K222R/K265R, 3KR). **(J)** Mapping the major site(s) for NleL-induced mono-ubiquitylation on JNK1. Flag-tagged wild-type JNK1 or its mutants bearing K-to-R substitution at each potential ubiquitylation site was individually transfected to HEK293T cells with His-NleL and HA-Ub-K29. **(K)** Lys-to-Arg substitution at Lys 68 (K68R) of JNK1 abolished NleL-induced mono-ubiquitylation of JNK1 in mammalian cells. **(L)** K68R of JNK1 abolished NleL-mediated JNK1 mono-ubiquitylation *in vitro*. Flag-tagged wild-type JNK1 or the indicated mutants (K68R and 3KR) were separately expressed in HEK293T cells and purified with anti-Flag M2 affinity beads. Purified JNK1 protein or its mutants was subjected to *in vitro* ubiquitylation reaction mixture at 37°C for 1h, followed by immunoblotting with indicated antibodies. All blots were representative of at least three independent experiments.

We next asked whether the interaction between NleL and host JNK might cause JNK ubiquitylation. As shown in Fig 2A, infection with wild-type EHEC O157:H7 increased the ubiquitylation of JNK, but infection with the Δ*nleL* strain had little or no impact on JNK ubiquitylation. Moreover, complementation of Δ*nleL* with wild-type NleL (but not the C753A mutant) effectively promoted JNK ubiquitylation in the infected cells. Thus, NleL could induce JNK ubiquitylation in the EHEC-infected host cells.

Although NleL_170-782_ was sufficient to mediate the assembly of poly-ubiquitin (poly-Ub) chains *in vitro* [15,16], the full-length form of NleL was used for all subsequent ubiquitylation assays and functional assays because of its stronger interaction with JNK1 and its intact E3 activity *in vitro* and *in vivo* (S2A and S2B Fig). Wild-type NleL, but not C753A, was found to efficiently promote mono- and poly-ubiquitylation of JNK1 *in vivo* and *in vitro*, depending on its E3 activity (Fig 2B–2D). Thus, these results established human JNK1 as the substrate for NleL.

NleL was previously shown to assemble Lys 6 and/or Lys 48 linked poly-Ub chains *in vitro*, while auto-ubiquitylation of NleL occurred preferentially via other Ub linkages [15,16]. Here, we found that NleL-catalyzed ubiquitin chains on JNK1 were primarily linked via Lys 27, Lys 29 and Lys 33 (K27, K29 and K33) linkages, especially the K29 linkage (S2C and S2D Fig). As shown in Fig 2E, NleL readily modified JNK1 with mono-Ub and K29-linked Ub chains, which can be completely removed by Usp2cc, the catalytic core of human ubiquitin-specific protease 2 (USP2). Our data also indicated that several E2s, particularly UbcH7, could support NleL-mediated JNK1 ubiquitylation in cells (S3A Fig).

### NleL promotes mono- and poly-ubiquitylation of JNK1 at different sites

We next mapped potential ubiquitylation sites in JNK1. JNK1α1, as the canonical isoform of human JNK1, contains 29 lysine residues. Trypsinolysis of ubiquitin conjugation yields signature “diGly remnants”, which could be enriched with anti-diGly monoclonal antibody for mass spectrometry (MS) analysis [24,25]. MS analysis of JNK1 purified from NleL-expressing 293T cells identified 6 diGly-containing peptides, which corresponded to ubiquitylation at Lys residues 68, 166, 250, 251, 265, and 308 (Fig 2F). The other five ubiquitylation sites in JNK1 (K140, K153, K203, K222, and K236) were identified by protein-protein docking analysis. Therefore, 11 Lys residues of JNK1 were the putative ubiquitylation sites for NleL (Fig 2G).

To further pinpoint the major Lys residues of JNK1 for NleL-induced ubiquitylation, *in vivo* ubiquitylation assays were performed with JNK1 mutants bearing Lys-to-Arg substitutions at each potential ubiquitylation site. Single Lys-to-Arg substitution on each of three sites (K153R, K222R or K265R) markedly attenuated NleL-induced JNK1 poly-ubiquitylation (Fig 2H and S3B and S3C Fig). Moreover, combined mutation of these three sites (K153/222/265R, 3KR) significantly abolished NleL-induced poly-ubiquitylation of JNK1 (Fig 2I). On the other hand, the K68R substitution alone completely abolished mono-ubiquitylation of JNK1 by NleL *in vivo* and *in vitro*, while the 3KR mutant was still clearly mono-ubiquitylated (Fig 2J–2L). Thus, four Lys residues (K68, K153, K222 and K265) of JNK1 were established as the major NleL-associated ubiquitylation sites, with the K68 residue predominantly responsible for mono-ubiquitylation.

### NleL-induced ubiquitylation of JNK1 abolishes its phosphorylation

**Fig 3:**
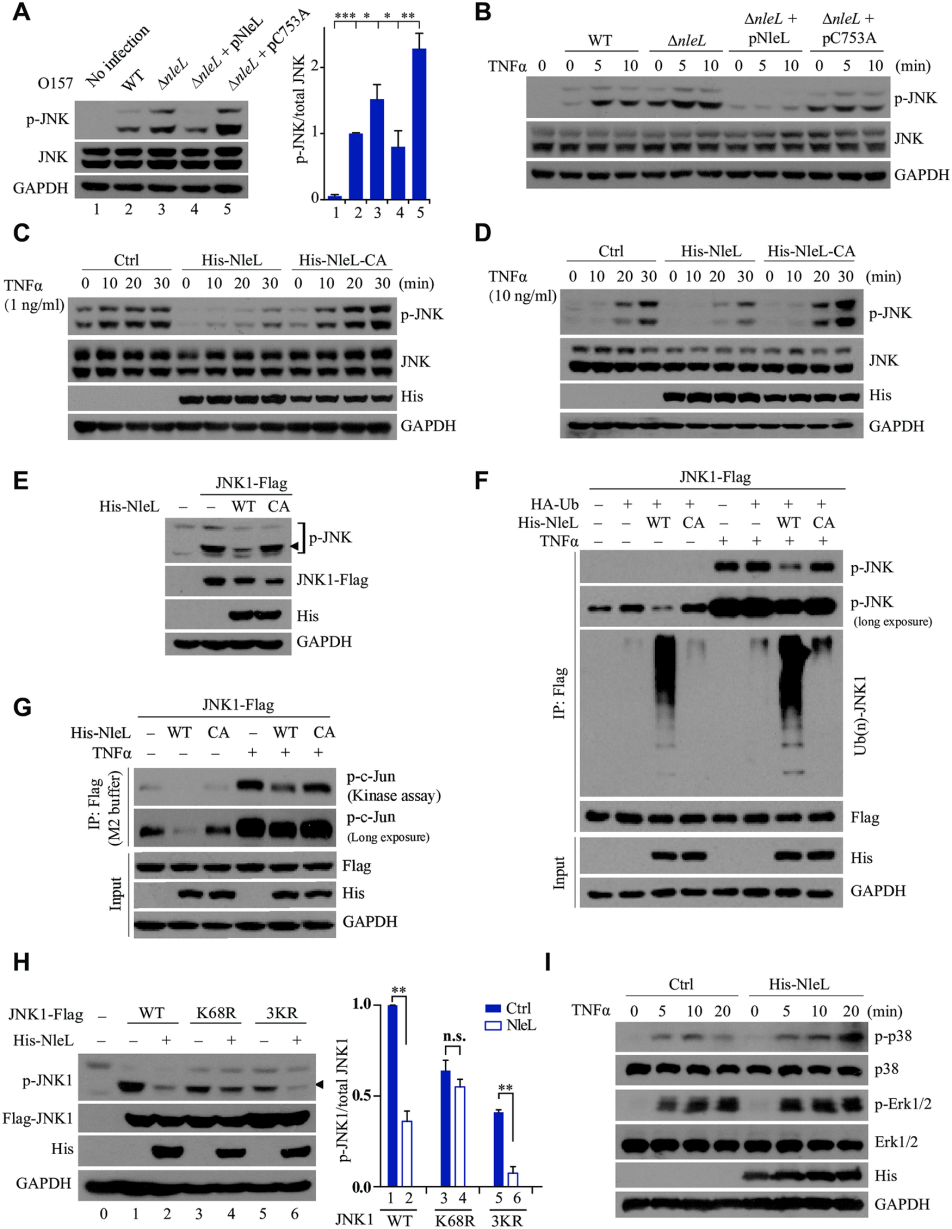
NleL-mediated JNK1 ubiquitylation inhibits phosphorylation of JNK1. **(A)** The Δ*nleL* strain of EHEC O157:H7 induced higher level of JNK phosphorylation than wild-type strain. Cells were infected with indicated EHEC strains with MOI of 100:1 for 2.5 h, and further sub-cultured 2 h in fresh DMEM medium. Then infected cells were lysed and subjected to IB analysis with indicated antibodies (left). The phosphorylated-JNK/ total JNK ratio (right) were calculated by quantifying protein bands. The lane numbers correspond to those shown in the left diagram. Bars represent means ± s.d., *P < 0.05, **P < 0.01, ***P < 0.001 (Student’s t-test, data are from three independent experiments, (n = 3)). **(B)** Cells were infected by indicated EHEC strains and then subjected to TNFα (10 ng/ml) treatment at the indicated time points. **(C** and **D)** NleL inhibited TNFα-mediated JNKs phosphorylation. HEK293T cells were transfected with plasmids encoding His-NleL or C753A mutant for 24 h. Cells were then stimulated by TNFα (1 ng/ml, **C** or 10 ng/ml, **D**). **(E)** Wild-type NleL, but not the C753A mutant, inhibited basal phosphorylation of ectopically expressed JNK1 in HEK293T cells. **(F)** NleL-induced JNK1 ubiquitylation conversely correlates with JNK1 phosphorylation. Indicated plasmids were transfected to HEK293T cells for 24 h. After transfection, cells were stimulated by TNFα (10 ng/ml) for 10 min and lysed in denatured RIPA buffer. Cell lysate was subjected to anti-Flag IP to enrich JNK1 protein. Phosphorylation and ubiquitylation status of JNK1 was assayed with indicated antibodies. **(G)** The E3 activity of NleL inhibits JNK activity. Plasmids expressing Flag–tagged JNK1 and His_6_-tagged NleL or its C753A mutant were co-transfected to HEK293T cells. 24 h after transfection, cells were treated with or without TNFα (10 ng/ml) for 10 min. Cells were then washed once in PBS and lysed in M2 buffer. Cell lysates were subjected to IP with anti-Flag M2 beads overnight. The anti-Flag M2 beads were then washed five times with M2 buffer, twice with 20mM Hepes (pH 7.4) and then subjected to the *in vitro* kinase assay, using GST-tagged c-Jun (1-79aa) as the substrate. The phosphorylation status of c-Jun was detected by anti-p-c-Jun antibody. **(H)** NleL did not suppress the TNFα-stimulated phosphorylation of K68R mutant of JNK1. HEK293T cells transiently expressed wild-type Flag-tagged JNK1 or its indicated mutants (K68R and 3KR). Cells were treated with TNFα (10 ng/ml) for 10 min. Phosphorylation of JNK1 was detected by immunoblotting analysis with anti-p-JNK antibody, and the phosphorylated-JNK1/ total JNK1 ratio (right) were further quantitated. The lane numbers correspond to those shown in the left panel. Bars represent means ± s.d., **P < 0.01, n.s., not significant. (Student’s t-test, data are from at least three independent experiments, (n = 3)). **(I)** NleL had no effect on TNFα-induced phosphorylation of p38 and Erk in mammalian cells. Cells were transfected with or without the plasmids encoding His_6_-tagged NleL for 24 h, and then subjected to TNFα treatment (10 ng/ml) at indicated times. Phosphorylation status of endogenous p38 or Erk was determined by IB with respective antibodies. Blots are representative of at least three independent experiments.

It has been established that activation of JNK signaling constitutes an early cellular response to bacterial infection [26,27]. We next investigated whether NleL functionally regulates this JNK role. As shown in Fig 3A and 3B, while wild-type EHEC induced slight phosphorylation of endogenous JNK in mammalian cells, infection by Δ*nleL* strain elicited much stronger JNK phosphorylation. Complementation of the Δ*nleL* strain with wild-type NleL restored the EHEC inhibitory effect on JNK phosphorylation, but the C753A-mutant–complemented Δ*nleL* strain did not (Fig 3A). Additionally, TNFα stimulation did not induce JNK phosphorylation when the mammalian cells were infected by the EHEC overexpressing wild-type NleL (but not C753A) (Fig 3B). These results prompted us to further investigate whether NleL alone is sufficient to suppress JNK phosphorylation.

Indeed, overexpression of wild-type NleL, but not NleL C753A, efficiently reduced the basal phosphorylation level of JNK (Fig 3C and 3E). Even when the cells were stimulated by TNFα at either low (1.0 ng/ml) or high (10.0 ng/ml) concentration, wild-type NleL significantly suppressed JNK phosphorylation (Fig 3C and 3D). Thus, the E3 ligase activity of NleL is sufficient to suppress JNK phosphorylation in host cells.

We further investigated the relevance of NleL-induced ubiquitylation to JNK1 phosphorylation. In the presence of wild-type NleL (but not C753A), JNK1 was ubiquitylated efficiently but poorly phosphorylated, suggesting that NleL-mediated JNK1 ubiquitylation might adversely impact JNK1 phosphorylation (Fig 3F). Moreover, a JNK kinase assay with recombinant c-Jun as the substrate revealed that the E3 activity of NleL markedly impaired the total kinase activity of JNK1 (Fig 3G). We then tried to explore the roles of JNK1 mono-ubiquitylation at the K68 residue and poly-ubiquitylation at other sites (K153, K222 and K265) in suppressing JNK1 phosphorylation. As shown in Fig 3H, NleL almost completely abolished TNFα-induced phosphorylation of wild-type JNK1 as well as the 3KR mutant; however, NleL had only negligible effects on the phosphorylation of the JNK1 K68R mutant. In addition, NleL had no effect on the phosphorylation of p38 or Erk, two other members of the MAPK superfamily (Fig 3I). Therefore, NleL-induced JNK1 ubiquitylation, particularly mono-ubiquitylation of K68, specifically inhibited JNK1 phosphorylation.

### NleL also targets other host JNK proteins (JNK2 and JNK3)

**Fig 4:**
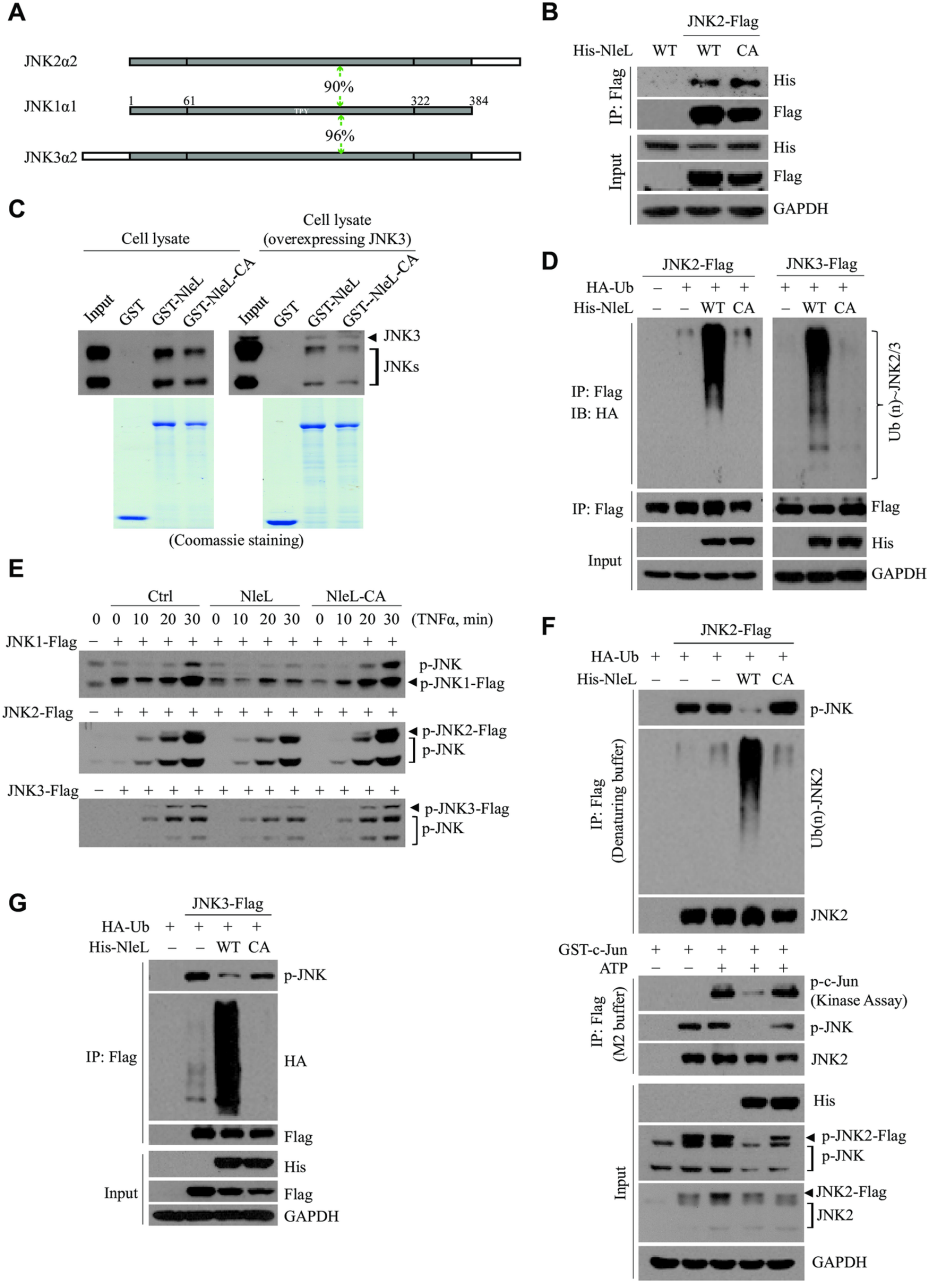
NleL targets other human JNK family proteins (JNK2 and JNK3). **(A)** A schematic view of the sequence homology between human JNK family proteins. The level of sequence homology between JNK1 and JNK2 or JNK3 (90% and 96%, respectively) is indicated. **(B)** NleL interacted with JNK2. Flag-tagged JNK2 and His_6_-tagged NleL (His-NleL) or C753A mutant (His-NleL-CA) were ectopically expressed in cells. Cells were lysed and subjected to Co-IP with anti-Flag beads, followed by IB analysis with indicated antibodies. **(C)** NleL interacted with endogenous JNKs or ectopically expressed JNK3. GST-tagged NleL, its C753A mutant or GST only was individually subjected to the GST pull-down assay with cell lysates of HEK293T cells transfected with (right) or without (left) Flag-tagged JNK3. The bound proteins were immunoblotted with anti-JNK antibody. **(D)** NleL promotes ubiquitylation of JNK2 (left) or JNK3 (right) *in vivo*. **(E)** NleL blocked TNFα-induced JNK1, JNK2 or JNK3 phosphorylation. HEK293T cells expressing Flag-tagged JNK1, JNK2 or JNK3 and His_6_-tagged wild-type NleL or its C753A mutant were subjected to TNFα treatment (10 ng/ml) at indicated times. Phosphorylation status of JNKs was determined by IB analyses with anti-p-JNK. **(F** and **G)** NleL-associated JNK2/3 ubiquitylation conversely correlates with the phosphorylation of JNK2/3. Phosphorylation and ubiquitylation status of JNK2 (**F**) or JNK3 (**G**) was assayed with Flag-JNKs enriched from cells expressing wild-type NleL or the C753A mutant, followed by immunoblotting analyses with indicated antibodies. The *in vitro* kinase assay was performed with Flag-tagged JNK2 (**F**). All the blots are representative of at least three independent experiments.

JNK family proteins share over 90% sequence homology to each other (Fig 4A and S4 Fig), including a conserved Lys 68 residue. We next asked whether NleL might also target JNK2 or JNK3, the other two members of the JNK family. Binding assays confirmed that NleL indeed interacted with JNK2 and JNK3 (Fig 4B and 4C). NleL, but not C753A, effectively catalyzed ubiquitylation of JNK2 and JNK3 (Fig 4D and S5A–S5C Fig), and inhibited their phosphorylation as well (Fig 4E and S5D Fig). Furthermore, similar to JNK1, ubiquitylation of JNK2/3 by NleL was negatively correlated with JNK2/3 phosphorylation and activation (Fig 4F and 4G). Thus, NleL appeared to target all the members of JNK family.

### NleL-induced ubiquitylation at Lys 68 residue of JNK suppresses the interaction between JNK and MKK7

**Fig 5:**
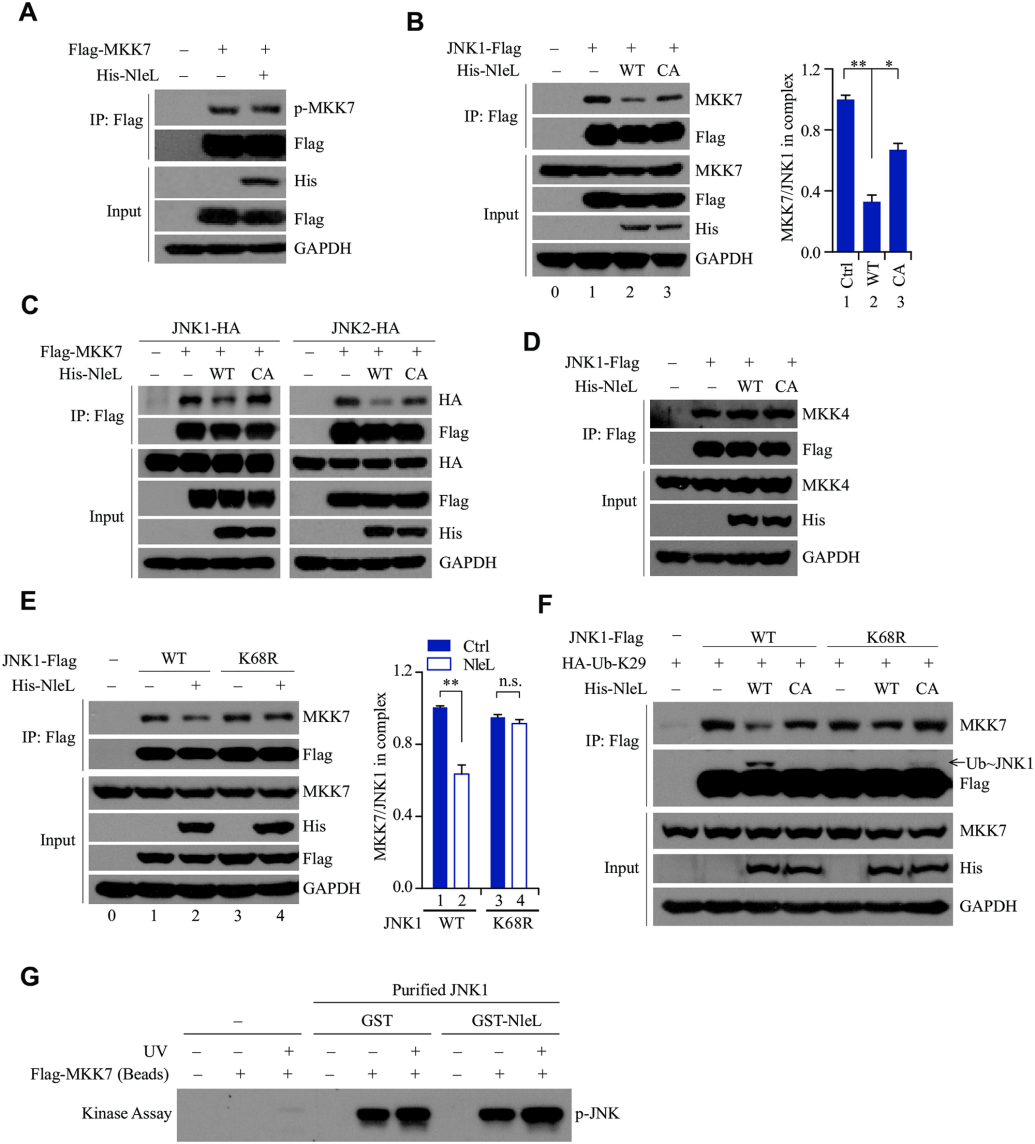
NleL mediated mono-ubiquitylation of JNK1 at Lys 68 inactivates JNK1 through disrupting the JNK1-MKK7 interaction. **(A)** NleL had little or no effect on MKK7 phosphorylation. HEK293T cells expressing Flag-tagged MKK7 and His-NleL were subjected to IP with anti-Flag, followed by IB with anti-p-MKK7 antibody. **(B)** NleL disrupted the interaction between JNK1 and endogenous MKK7. HEK293T cells expressing Flag-tagged JNK1 and His_6_-tagged NleL or the C753A mutant were lysed 24 h after transfection. Co-immunoprecipitation with anti-Flag M2 beads and immunoblotting with anti-MKK7 antibody were performed (left). The MKK7/JNK1 ratios in immunoprecipitate complexes were further determined by quantifying protein bands (right). **(C)** Co-immunoprecipitation assays of ectopically expressed Flag-tagged MKK7 and HA-tagged JNK1 (left) or JNK2 (right) in the cells expressing His-NleL or C753A. The immuoprecipitates and whole-cell lysates (input) were subjected to immunoblotting with the indicated antibodies. **(D)** NleL did not disrupt the interaction between JNK1 and MKK4. **(E)** K68R of JNK1 almost totally abolished the NleL-mediated disruption of the JNK1-MKK7 interaction. HEK293T cells transiently expressed wild-type Flag-tagged JNK1 or the K68R mutant with or without His_6_-tagged NleL. Cells were treated with TNFα (10 ng/ml) for 10 min before being lysed. Then immunoprecipitation with anti-Flag M2 beads and subsequent immunoblotting with anti-MKK7 antibody were performed. The MKK7/JNK1 ratio in immunoprecipitates (right) were further determined by quantifying the densities of the concerned protein bands. **(F)** NleL suppressed the interaction between JNK1 and MKK7 by promoting mono-ubiquitylation on Lys 68 of JNK1. HEK293T cells were transfected with indicated plasmids for 24 h, following by TNFα (10 ng/ml) stimulation for 10 min. **(G)** Co-expression of GST-tagged NleL and His_6_-tagged JNK1 in bacteria showed no effect on JNK1 phosphorylation by MKK7 *in vitro*. His_6_-tagged JNK1 was purified by using Ni-NTA beads from *E*. *coli* BL21 (DE3) strain co-expressing His_6_-tagged JNK1 and GST-tagged NleL or GST only. HEK293T cells expressing Flag-tagged MKK7 (with or without UV stimulation) were lysed in M2 buffer and subjected to anti-Flag immunoprecipitation. Then the beads bounded with Flag-tagged MKK7 were subjected to *in vitro* kinase assay with bacterially purified His_6_-tagged JNK1 as the substrate, followed by IB analysis with anti-p-JNK antibody. In **(B)**, **(E)**, Bars represent means ± s.d. from three biological replicates, *P < 0.05, **P < 0.01, n.s., not significant. (Student’s *t*-test, data are from three independent experiments, (n = 3)). Blots are representative of at least three independent experiments.

Currently, two MAP2Ks, MKK4 and MMK7, are known to phosphorylate and activate JNK proteins, with MKK7 being more specific to JNK [28]. It is natural to speculate that NleL-induced ubiquitylation might suppress JNK phosphorylation by either inhibiting the kinase activity of MKK7 or disrupting the interaction between MKK7 and JNK. Since NleL had little or no effect on the phosphorylation of MKK7 (Fig 5A), we proceeded to explore the latter possibility. Usually, JNK1 or JNK2 interacts with MKK7 in host cells. NleL readily reduced MKK7 association with JNKs, but the C753A mutant had little effect (Fig 5B and 5C and S6A and S6B Fig), suggesting that NleL impairs the MKK7-JNK interaction independent of direct competition against MKK7. We also ruled out the possibility that NleL might obstruct MKK4 phosphorylation or MKK4-JNK association (Fig 5D and S6C Fig). Moreover, NleL disrupted the recruitment of wild-type JNK1 to MKK7, but had no effect on the interaction of K68R mutant with MKK7, although wild-type JNK1 and its K68R mutant had the same ability to interact with MKK7 (Fig 5E and 5F). Thus, the K68 residue of JNK1 is required for NleL to suppress the MKK7-JNK interaction.

Since *E*. *coli* do not have the ubiquitin system, NleL should not conjugate Ub to JNK1 in bacterial cells. If NleL could mediate modifications in addition to ubiquitylation, it could still potentially modify and inactivate JNK1 when co-expressed with JNK1 in *E*.*coli*. However, we found that MKK7 readily phosphorylated purified JNK1 that was co-expressed with either GST-tagged NleL or GST alone in the *E*. *coli* BL21 (DE3) strain (Fig 5G). These data suggested that NleL did not inactivate JNK1 through other post-translational modifications.

Altogether, we conclude that NleL-induced ubiquitylation at the K68 residue of JNK1 suppresses the phosphorylation of JNK1, through disrupting the JNK1-MKK7 interaction.

### NleL-induced JNK ubiquitylation impairs host JNK/AP-1 signaling

**Fig 6:**
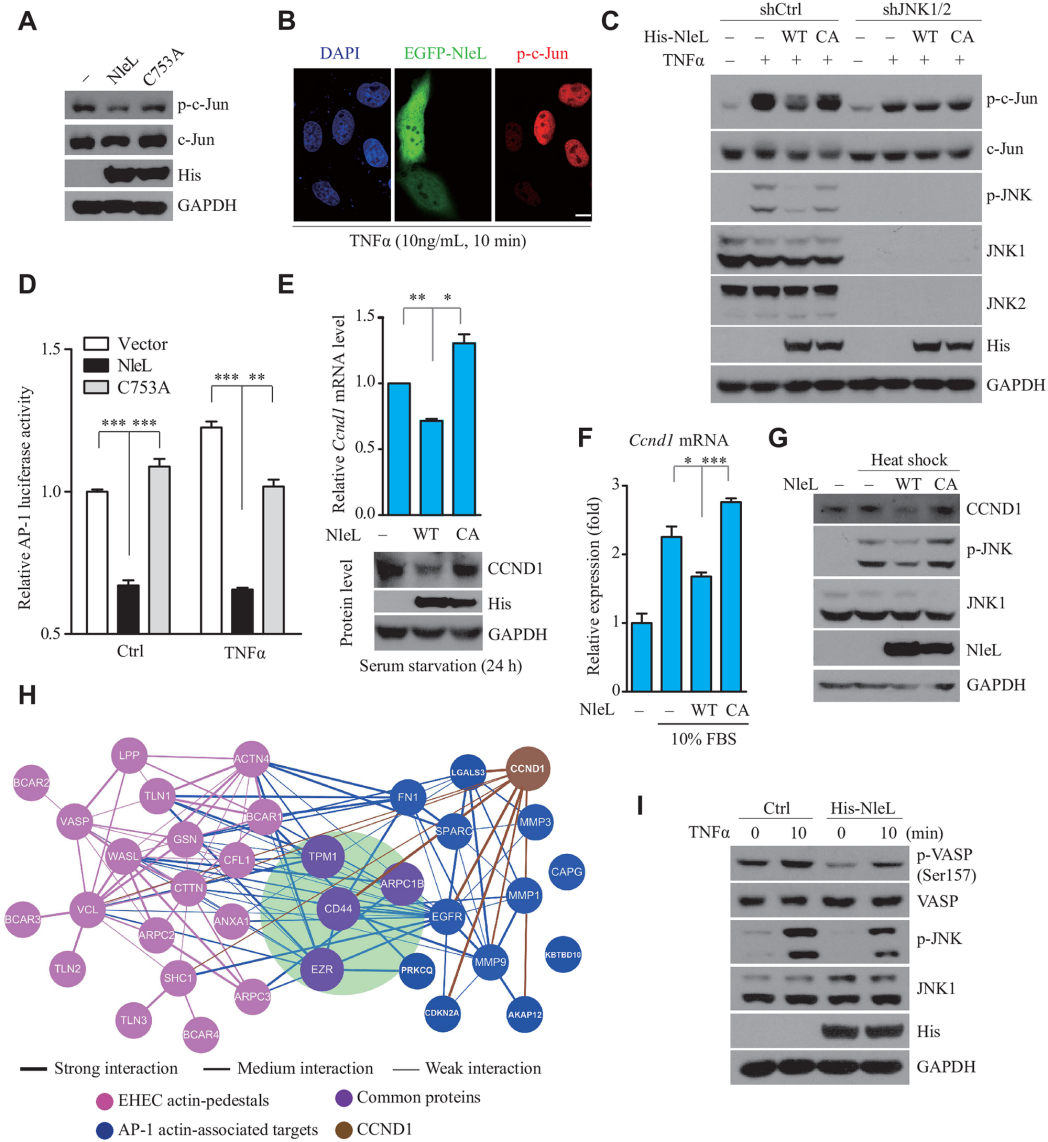
NleL-mediated JNK ubiquitylation disrupts the JNK/AP-1 signaling pathway in the mammalian cells. **(A)** NleL inhibited the basal-level phosphorylation of c-Jun in HEK293T cells. **(B)** NleL prevented the activation of endogenous c-Jun upon TNFα stimulation. Cells were transfected with EGFP-NleL for 24 h before TNFα stimulation (10 ng/ml). Immunofluorescence microscopy analysis was performed with anti-phosphorylated c-Jun antibody (p-c-Jun, in red). Nuclei were counterstained with DAPI (blue). Scale bars represent 10 μm. Shown are representative images from three independent experiments. **(C)** *Jnk1/2* knockdown blocked NleL-mediated inactivation of c-Jun. Cells stably expressing control shRNA (shCtrl) or shRNA for *Jnk1/2* (shJNK1/2) were transfected with indicated plasmids for 24 h before TNFα stimulation (10 ng/ml, 10min). Then phosphorylation level of c-Jun and JNK were determined by IB analyses. **(D)** NleL suppressed the transcription factor activity of AP-1. HEK293T cells expressing wild-type NleL or its C753A mutant were co-transfected with the dual luciferase AP-1 reporter system. Twenty-four hours after transfection, the cells were stimulated by TNFα (10 ng/mL, 6 h) and then subjected to a luciferase activity assay. Data are represented as the mean ± s.d. from three biological replicates, ***P<0.001. (Student’s *t*-test, data from three biological replicates (n = 3)). **(E)** NleL down-regulated the homeostatic level of endogenous cyclin D1 (CCND1) at mRNA (upside) or protein (downside) level. Data are represented as the mean ± s.d from three biological replicates, *P < 0.05, **P < 0.01. (Student’s *t*-test). **(F)** NleL reduced 10% FBS-stimulated transcription of cyclin D1. HEK293T cells expressing NleL or C753A mutant were serum-starved for 24 h and then stimulated with 10% FBS. Then qRT-PCR was performed to determine cyclin D1 expression at mRNA level. Data are shown as the mean ± s.d. from three biological replicates, *P < 0.05, ***P < 0.001 (Student’s *t*-test, n = 3). **(G)** NleL suppressed the CCND1 expression up-regulated by heat shock. Cells expressing NleL or C753A were serum-starved for 24 h, and then subjected to heat shock (42°C, 40 min). **(H)** Protein-protein interactions among the actin-associated proteins targeted by transcription factor AP-1 and proteins characterized in EHEC actin pedestals (blue and pink, respectively). Hits common in both groups are shown in purple, with the interactions directly related to AP-1 targets shown in blue and the interactions only among actin-pedestal proteins in pink. And, CCND1 and its interaction are brown. Line thickness indicates the interaction level of proteins. **(I)** NleL regulated phosphorylation of an actin-pedestal protein VASP. HEK293T cells transfected with or without a plasmid expressing His_6_-tagged NleL were stimulated by TNFα (10 ng/ml, 10 min), and then subjected to IB analyses with indicated antibodies. Blots are representative of at least three independent experiments.

We found that NleL-mediated JNK inactivation was independent of NF-κB signaling (S7A Fig). Next, we examined the possible effects of NleL on downstream of JNK. As expected, wild-type NleL suppressed the basal-level phosphorylation of endogenous c-Jun, a *bona fide* physiological substrate of JNK, while the C753A mutant did not (Fig 6A). Immunofluorescence microscopy analysis also revealed that EGFP-tagged NleL, but not EGFP, markedly reduced c-Jun phosphorylation (Fig 6B and S7B Fig). Consistently, overexpression of NleL (but not C753A) impaired TNFα-stimulated phosphorylation of c-Jun (Fig 6C). However, depletion of *Jnk1/2* using short hairpin RNAs in mammalian cells almost completely abolished the inhibitory effect of NleL on c-Jun phosphorylation (Fig 6C). Therefore, NleL inhibits c-Jun phosphorylation by targeting JNK proteins.

As c-Jun is a major component of the AP-1 transcription factor [29], we next investigated the regulation of AP-1 activity by NleL. An AP-1 luciferase reporter assay showed that wild-type NleL, but not C753A, suppressed AP-1 activity in cells (Fig 6D and S7C and S7D Fig). AP-1 is known to regulate the expression of a large number of genes, *e*.*g*. cyclin D1 (CCND1) [30]. As expected, the basal expression of CCND1 in mammalian cells was down-regulated by NleL (but not C753A) (Fig 6E). Consistently, NleL also diminished JNK phosphorylation and CCND1 expression induced by different stimulators (Fig 6F and 6G and S7E and S7F Fig). These results suggest that the E3 activity of NleL is required to suppress AP-1 activity and the expression of AP-1 target genes.

Based on previous work by multiple groups [4,5,31], some AP-1 targets (*e*.*g*. CD44, TPM1, ARPC1B and EZR) are known to be important for the formation of EHEC-induced pedestals. A protein-protein interaction (PPI) network analysis was performed to characterize the potential interplay among the actin-associated proteins targeted by AP-1 and the host proteins identified in EHEC actin pedestals (Fig 6H). The data strongly suggested an emerging role of AP-1 signaling in regulating the formation of EHEC actin pedestals. Furthermore, we found that ectopically expressed NleL suppressed the phosphorylation of VSAP (Fig 6I), one of the critical components in actin pedestals that was recently reported to be modulated by JNK/AP-1 signaling [32]. Meanwhile, CCND1, another AP-1 target protein shown above to be down-regulated by NleL, also interacts with AP-1 actin-associated targets and EHEC pedestal proteins (Fig 6H). Thus, NleL might promote the formation of EHEC actin pedestals by modulating the host JNK/AP-1 pathway.

### JNKs are the major targets of NleL in promoting EHEC-induced A/E lesions and EHEC infection

**Fig 7:**
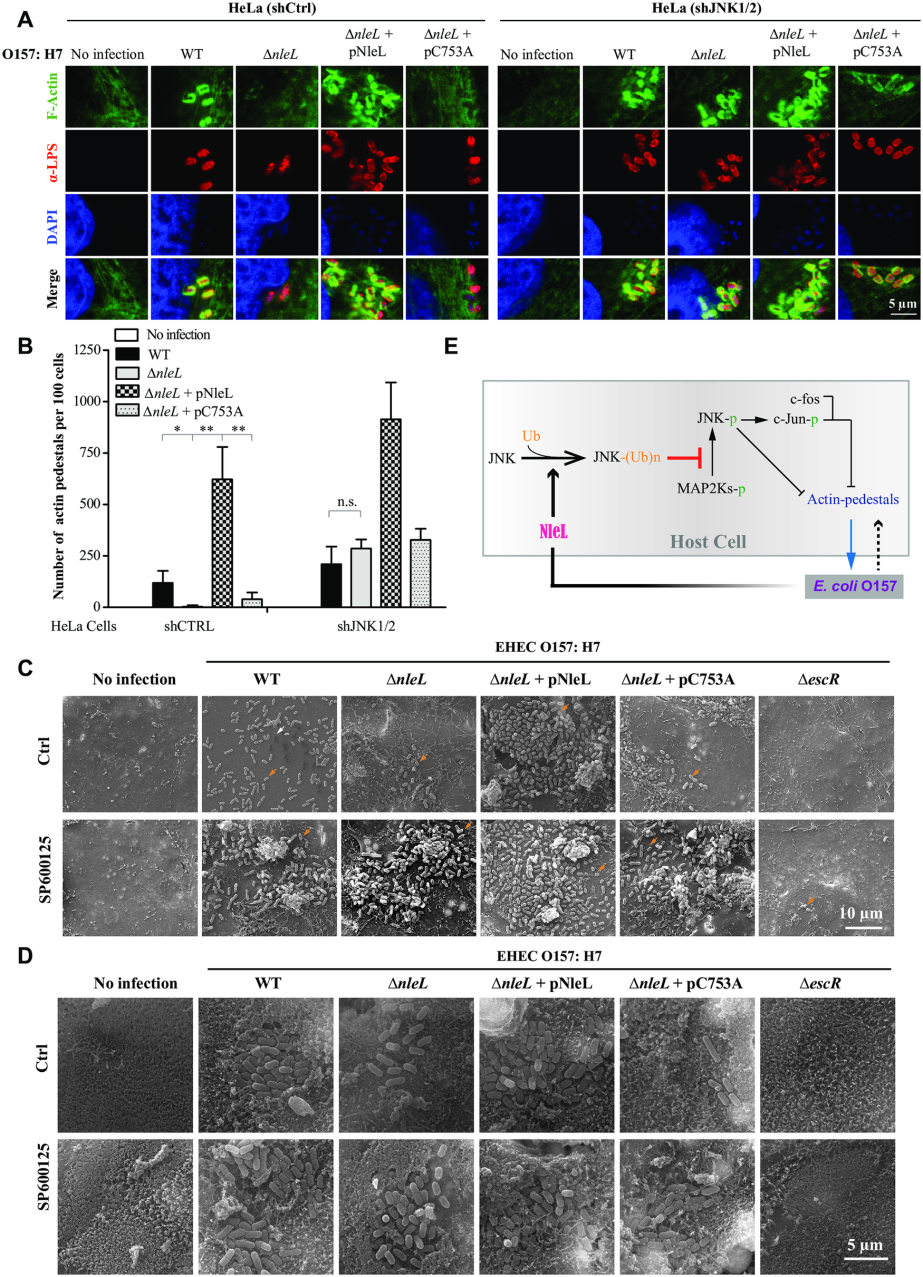
NleL promotes the ability of EHEC O157:H7 to cause A/E lesions on mammalian cells by inhibiting JNK activation. **(A** and **B)** NleL promoted the formation of actin pedestals induced by EHEC O157:H7. HeLa cells stably expressing control shRNA (shCtrl) or shRNA for *Jnk1/2* (shJNK1/2) were co-cultured with indicated EHEC strains for 2 h, then washed with PBS and further cultured for 2.5 h in the fresh medium. After infection, cells were subjected to immunofluorescence microscopy analysis. Shown are representative cell images where anti-*E*. *coli* LPS staining indicates bacteria (red), DAPI staining marks the nucleus (blue) and F-actin denotes the filamentous actin stained by CytoPainter Phalloidin-iFluor 488 Reagent (green) (**A**). Shown in (**B**) are numbers of actin pedestals per 100 cells. More than 100 infected cells were examined for each infection experiment and data are represented as mean ± s.d. from three independent experiments. Statistical significance was determined by Student’s *t*-test. *P < 0.05, **P < 0.01, n.s., not significant. **(C)** NleL enhances the ability of EHEC O157:H7 to infect Caco-2 monolayer (grown for 6 days) by targeting host JNKs. Caco-2 monolayers (grown for 6 days) treated with DMSO or JNK inhibitor SP600125 (10 μM) were infected with EHEC strains for 2.5 h, then washed with PBS and further cultured for 4 h in fresh medium. After infection, cells were subjected to scanning electron microscopy (SEM) analyses. The white arrow represents an actin pedestal; the orange arrows represent bacteria. **(D)** NleL promotes the ability of EHEC O157:H7 to form A/E lesions on Caco-2 monolayers (grown for 21 days) by inhibiting JNK proteins. Caco-2 monolayers (grown for 21 days) treated with DMSO or JNK inhibitor SP600125 (10 μM) were infected with EHEC strains for 2.5 h, then washed with PBS and further cultured for 4 h in fresh medium. After infection, cells were subjected to SEM analyses. **(E)** A schematic diagram showing how NleL-mediated JNK ubiquitylation promotes A/E lesion and EHEC colonization through suppressing JNK phosphorylation and impairing MKK7/JNK/AP-1 signaling.

We next explored the role of NleL on the formation of EHEC actin pedestals. Compared to the wild-type EHEC strain, deletion of *nleL* from EHEC reduced actin-pedestal formation on mammalian cells (Fig 7A). Complementation of Δ*nleL* strain with NleL, but not C753A, significantly restored the EHEC pedestal-forming abilities (Fig 7A and 7B). On the other hand, *Jnk1/2* depletion in HeLa cells promoted actin-pedestal formation by each of the EHEC strains. Additionally, Δ*nleL* and wild-type EHEC had similar pedestal-forming abilities on *Jnk1/2*-silenced host cells (Fig 7A and 7B). Thus, NleL promoted the formation of EHEC actin pedestals through targeting host JNKs.

*C*. *rodentium* has been used as an alternative approach to study EHEC. However, differing from our findings on the role of NleL in EHEC infection, NleL-deficiency was found to have no effect on the ability of *C*. *rodentium* to form actin pedestals and attach to HeLa cells or mice colon (S8 and S9 Fig). Instead, overexpression of EHEC NleL in the Δ*nleL C*. *rodentium* suppressed the ability of *C*. *rodentium* to attach to HeLa cells and form actin pedestals. Thus, *C*. *rodentium* was not a suitable model system to study NleL.

As an A/E pathogen, the pedestal-forming ability of EHEC is considered to correlate with its capability to colonize host [4]. Caco-2 is a human colorectal epithelial cell line that can form an epithelial cell monolayer when cultured for 6 days; continued Caco-2 monolayers growth for 21 days can become differentiated with a brush border that more closely resembles human intestinal epithelium [33–35]. We next performed EHEC infection assays on 6-day-old and 21-day-old Caco-2 monolayers. NleL increased the ability of EHEC to colonize Caco-2 monolayers in a manner dependent on NleL’s E3 ligase activity. Moreover, treatment of Caco-2 with the JNK inhibitor SP600125 effectively rescued the capability of the Δ*nleL* strain to attach to the Caco-2 monolayer (Fig 7C and S10A and S10B Fig). Similar results were also observed in HeLa cells (S11 Fig). We additionally observed EHEC-induced A/E lesions on 21-day-old Caco-2 monolayers (Fig 7D). Wild-type EHEC (but not Δ*nleL*) caused A/E lesions on 21-day-old Caco-2 monolayers, marked by microvillus damage. Complementation of the Δ*nleL* strain with wild-type NleL, but not the C753A mutant, restored the EHEC A/E lesion-forming ability; SP600125 treatment also rescued the ability of Δ*nleL* to form A/E lesions on the Caco-2 monolayer. These data suggested that the suppression of JNK1/2 functions in host cells could compensate for *nleL*-deletion-caused loss of the EHEC ability to colonize the host and form A/E lesions. JNK proteins are thus identified as the critical host targets for NleL to promote EHEC O157:H7 infection (Fig 7E).

## Discussion

In this work, we have demonstrated that NleL, a bacterial effector and HECT-like E3 ubiquitin ligase from EHEC O157:H7, is critically involved in promoting actin-pedestal formation during EHEC infection. Notably, this finding is different from a previous report by Piscatelli *et al*., in which NleL was shown to down-regulate the EHEC-induced formation of actin pedestals [19]. If NleL down-regulates actin-pedestal formation in infection as suggested by Piscatelli *et al*., the presence of NleL should have disrupted bacterial attachment to the host cells. However, results from the same study by Piscatelli *et al*. indicated that EHEC NleL, reintroduced into the *nleL*-deleted *C*. *rodentium*, was actually required for efficient infection *in vivo*. Further work is needed to clearly understand the discrepancy between our findings and the findings of Piscatelli *et al*.

As described above, *C*. *rodentium* cannot be a suitable surrogate for EHEC O157:H7 to study NleL. Multiple studies have indicated that EHEC can trigger actin-pedestal formation in host cells in ways differing from *C*. *rodentium* [36–38]. While EHEC O157 uses the bacterial effector Tir and TccP adaptor protein to trigger actin polymerization, *C*. *rodentium* relies on the phosphorylation of Tir Y471 and the host protein Nck. This may partially account for why the NleLs from these two pathogens have very different effects on actin-pedestal formation, despite their high homology. Given that EHEC NleL targets and suppresses the JNK/AP-1 pathway, it is likely that NleL plays roles in the later stages, rather than the triggering stage, of actin polymerization. Further work is warranted to elucidate the mechanisms underlying why NleL has different roles in these two bacteria. On the other hand, we found that the differentiated Caco-2 monolayers (grown for 21 days) could be used as an *in vitro* infection model to study A/E lesions. Caco-2 monolayers can mimic human colonic epithelium for EHEC infection, providing an alternative approach to *in vivo* infection.

Different Ub chain linkages have different impacts on targeted proteins (e.g. K48- or K63-linked Ub chains usually cause protein degradation) [39–41]. Here, NleL primarily assembled mono-Ub and K29-linked Ub chains on JNK, suggesting a non-proteolytic function of NleL-induced JNK ubiquitylation. Instead, NleL-induced mono-ubiquitylation at K68 of JNK, but not the poly-ubiquitylation at other residues, disrupted the interaction between JNK and MKK7. Although a recent report showed that JNK1 binds MKK7 using multiple binding sites [42], K68 of JNK1 is not in the MKK7-JNK1 binding interface. Other mechanisms, such as those involving allostery, may underlie how JNK1 mono-ubiquitylation disrupts the MKK7-JNK1 interaction.

NleL-mediated JNK mono-ubiquitination appeared to drastically suppress JNK activity, although the mono-ubiquitylated sub-population of JNK1 seemed limited (5% ~ 10% of all cellular JNK1). Currently there are two accepted explanations: 1) cellular proteins can exist in different subcellular populations, so targeted ubiquitylation of a particular subpopulation can be sufficient to generate significant impacts on a specific pathway; 2) NleL-induced JNK1 ubiquitylation should be a dynamic process balanced by removal of ubiquitin by deubiquitylating enzymes (DUBs), as there are plenty of DUBs in mammalian cells [43–45]. In other words, although only part of JNK were observed to be modified by NleL, it is highly possible that most of the JNK molecules undergo NleL-mediated ubiquitylation and then deubiquitylation by DUBs.

The JNKs are master regulators in mammalian cells [46]. It is well established that JNKs are phosphorylated by upstream kinases and then activate downstream targets. However, little was known about the posttranslational modifications other than phosphorylation that might occur on JNKs until recently, when several ubiquitylation and acetylation sites of endogenous JNKs were uncovered by proteomic analyses [24,47,48]. Whether these potential modifications might impact the functions of JNKs remains poorly understood. We thus demonstrate for the first time that ubiquitylation of JNKs by the bacterial effector NleL negatively regulates the function of JNKs. It will be intriguing to investigate whether an unknown endogenous E3 ligase might exist to catalyze the ubiquitylation of JNKs and regulate JNK signaling in mammalian cells.

Worldwide, outbreaks of EHEC O157:H7 infection constitute constant and serious threats to human population and live stocks, without effective treatments [49]. Antibiotic therapy is generally contraindicated as it may promote expression of Stx toxin protein and increase the risk of the hemolytic–uremic syndrome (HUS) [50–52]. Only supportive care can be provided for the infected patients who have developed HUS. Thus, a diversity of treatment and prevention strategies should be developed to protect against EHEC. As NleL promotes EHEC infection by suppressing host JNK, disrupting the NleL-JNK interaction may represent a novel strategy against EHEC O157:H7 infections.

## Materials and methods

### Ethics statement

All animal use procedures were in strict accordance with the Guide for the Care and Use of Laboratory Animals (8th edition, National Research Council, 2011), approved by the Institutional Animal Care and Use Committee (Protocol number SIBCB-S330-1512) of Shanghai Institute of Biochemistry and Cell Biology, Chinese Academy of Sciences.

### Plasmids, antibodies, and reagent

DNAs for NleL amplified from the genomic DNA of *E*. *coli* O157:H7 Sakai strain was inserted into pCDNA3.0 and pEGFP-C1 for mammalian expression, and pGEX-4T-1 for recombinant expression in *E*. *coli*. NleL DNA was also ligated into the pTRC99A vector for complementation in EHEC (under the trc promoter; pTRC99A is kindly provided by Dr. Xueli Zhang from Tianjin institute of industrial biotechnology, Chinese academy of sciences). Genes for encoding JNK1α1, JNK2α2 (kindly provided by Dr. Jinzhang Zeng from Xiamen University, China), JNK3α2 (Addgene #13759) and MKK7 (Addgene #14623) were cloned to pCDNA3.0 vector with a C-terminal Flag tag (or HA tag), and pET28a vector with 6× His tag. AP-1 luciferase reporter plasmid was a gift from Dr. Jine Yang (Sun Yat-sen University, Guangzhou, China). Plasmids expressing HA-tagged Ub and its mutants were described previously [53]. All the point mutations were generated by using the QuickChange Site-Directed Mutagenesis Kit (Stratagene) according to manufacturer’s protocol. All constructs were verified by DNA sequencing.

Antibodies for JNK1 (2C6) (#3708), phospho-SAPK/JNK (81E11) (#4668), phospho-SAPK/JNK (G9) (#9255), phospho-c-Jun (54B3) (#2361), phospho-c-Jun (#9261), caspase-3 (#9662), VASP (9A2) (#3132) and phospho-VASP (Ser157) were obtained from Cell Signaling Technology. Anti-JNK2 antibody (EP1595Y) (ab76125), anti-MEK7 antibody (EP1455Y) (ab52618), anti-JNK1/2/3 antibody (ab179461) and anti-MEK7 (phospho S271 + T275) (ab4762) were purchased from Abcam. Rabbit anti-HA antibody (H6908), mouse monoclonal anti-Flag antibody (F1804), and Anti-Flag M2 affinity gel (A2220) were from Sigma. Other antibodies were purchased from BD pharmingen for anti-JNK1 (551197), Santa Cruz Biotechnology for anti-ubiquitin (P4D1), Bioword for anti-c-Jun (G237), Absci for anti-MKK4 (#AB21132) and anti-phospho-MKK4 (Ser80) (#AB11177), HangZhou HuaAn Biotechnology for anti-His_6_ tag (M0812-3), Proteintech for mouse anti-Cyclin D1 (60186-1-Ig), rabbit anti-Flag tag (20543-1-AP) and mouse anti-GAPDH (60004-1-Ig). Peroxidase-conjugated goat anti-rabbit and goat anti-mouse IgG secondary antibodies were purchased from Jackson ImmunoResearch Laboratories, Inc. Chemicals were purchased from Sigma if not otherwise indicated: PS-1145 (Santa Cruz Biotechnology), ATP (Thermo Scientific Fermentas). JNK inhibitor SP600125 (S1460) was from Selleck. HeLa, Caco-2 and HEK293T cells were obtained from the American Type Culture Collection (ATCC). All cell culture products were from Corning.

### Cell culture and transfection

HEK293T, HeLa, Caco-2 cells were cultured in Dulbecco’s modified Eagle’s medium (DMEM) (Hyclone) supplemented with 10% fetal bovine serum (FBS), 2.0 mM L-glutamine, 100 units/ml penicillin and 100 mg/ml streptomycin. Cells were maintained in 5.0% CO_2_ at 37°C. Transfections were carried out with Lipofectamine 2000 (Invitrogen) according to the manufacturer’s instruction.

### Bacteria culture and manipulation

Enterohemorrhagic *Escherichia coli* (EHEC) O157:H7 Sakai strain (RIMD 0509952) and *C*. *rodentium* (CR) strain DBS100 (ATCC 51459) were used as wild-type strains. These bacteria were commonly cultured at 37°C LB broth. Deletion of *nleL* from EHEC O157:H7 genome was achieved through standard homologous recombination, as reported previously [22]. For CR, the *nleL*-deleted mutant and *escR*-deleted mutant had been constructed through a homologous recombination method “Gene doctoring” [54,55]. The mutants were verified by PCR and DNA sequencing. For rescue assay, the Δ*nleL* strain was transformed with plasmid encoding wild-type Flag-tagged NleL or its C753A mutant.

### Bacterial infection of mammalian cells

The infection was performed as described before with slight modifications [56,57]. Briefly, EHEC or CR strains were cultured overnight in 2 × YT (16.0 g/L tryptone, 10.0 g/L yeast extract, 5.0 g/L NaCl) medium without shaking at 37°C. Bacterial cultures were then diluted by 1:40 with serum-free DMEM medium, and cultured for an additional 3 ~ 4 h at 37°C in the presence of 5% CO_2_ to induce the expression of type III secretion system before infection. For the complementation assay in EHEC or CR, the medium was added with 1.0 mM Isopropyl-B-D-thiogalactopyranoside (IPTG). Bacterial cells were then collected and suspended in PBS. After measuring the O.D. of cultured bacteria, infections were performed with mammalian cells at a multiplicity of infection (MOI) of 100:1 or 20:1, if not indicated otherwise, with a centrifugation at 800 g for 10 min, and then proceeded with incubation at 37°C in 5% CO_2_ for 2 ~ 3 h. After that, cells were washed three times and further cultured in fresh DMEM medium for another 2.5 ~ 5 h. Then the infected cells were subjected to immunofluorescence assay or IB analyses with indicated antibodies.

### Expression and purification of recombinant proteins

*E*. *coli* BL21 (DE3) strains harboring the corresponding recombinant plasmids were grown in LB medium supplemented with appropriate antibiotics. Protein expression was induced overnight at 16°C with 0.3 mM IPTG when OD600 reached 0.6 ~ 0.8. To purify His_6_-tagged proteins, bacteria were harvested and lysed in lysis buffer containing 50mM Tris-HCl (pH 7.6), 300 mM NaCl, 40 mM imidazole and 5.0 mM beta-mercaptoethanol, and then proteins were purified with affinity chromatography using Ni-NTA beads (Qiagen) according to the manufacturer’s instruction. For GST-fusion proteins, purifications were performed by affinity chromatography using Glutathione Sepharose Fast Flow beads (GE Healthcare). Eluted proteins were further dialyzed overnight at 4°C. Recombinant proteins were concentrated and then frozen-stored in a buffer containing 50 mM Tris-HCl (pH 7.4), 300 mM NaCl and 15% Glycerol (V/V). Protein concentrations were determined using Bradford colorimetric assays, with their protein purities examined with SDS-PAGE followed by Coomassie Blue staining.

### Gene knockdown

Nucleotide sequences for the human *Jnk1/2*-specific shRNAs used were described before [58]. For stable knockdown of *Jnk1/2*, lentiviral particles harboring specific shRNA expression vector (pLKO.1; Sigma-Aldrich) were produced by transfection of HEK293FT cells with the shRNA expression plasmid and lentiviral packaging mix. Target cells (HEK293T and HeLa) were incubated with the viral supernatant in the presence of 8 μg/ml polybrene (Sigma) and selected with 2 μg/ml puromycin (Clontech).

### *In vitro* ubiquitylation assay

As previously described with minor modifications [59], *in vitro* ubiquitylation assays were carried out in a 30 μl reaction system containing E1 (100 ng), His_6_-tagged UbcH7 (200 ng), GST-NleL or C753A (500 ng), Flag-JNK1 (500 ng) and ubiquitin (1.0 μg) in ubiquitylation buffer (50 mM Tris–HCl, pH 7.5, 5.0 mM MgCl_2_, 2.0 mM ATP, 1.0 mM DTT) at 37°C for 60 min. After the reaction was terminated by adding one-tenth volume of 10 × SDS sample buffer, the resulted mixtures were boiled and then subjected to SDS-PAGE, followed by IB analysis with indicated antibodies.

### *In vivo* ubiquitylation assay

*In vivo* ubiquitylation were also carried out as described previously [60]. Cells were collected and lysed in denaturing RIPA buffer containing 50 mM Tris-HCl (pH 7.6), 150 mM NaCl, 1.0% Triton X-100, 1.0% sodium deoxycholate and 0.1% SDS supplemented with 1.0% protease inhibitor cocktail (Roche). Cell lysates were then centrifuged at 15,000 g for 10 min, 4°C. Following overnight incubation of anti-Flag beads with cell lysate at 4°C, the beads was washed five times with RIPA buffer. Finally the beads were boiled in SDS sample buffer and subjected to IB analysis with indicated antibodies.

### Immunoprecipitation

HEK293T cells in 6 cm dish were transfected with 5.0 μg pCDNA3-Flag-JNK1 and cultured for 24 h. Cells were then lysed in IP buffer (containing 50 mM Tris-HCl, pH 7.6, 150 mM NaCl, 1.0% Triton X-100), and centrifuged (15,000g) for 15 min at 4°C. The supernatants were then subjected to IP with anti-Flag M2 beads to enrich Flag-JNK1. Then Flag-JNK1 was eluted by using 3 × Flag peptides (150 μg/ml in PBS) at 4°C for 30min. The eluted protein was verified by Coomassie Blue staining of SDS-PAGE gels.

### Co-immunoprecipitation (Co-IP) and GST pull-down assay

For Co-IP assay, 24 h after transfection with indicated plasmids, HEK293T cells were lysed in Triton X-100 buffer (50 mM Tris-HCl at pH 7.4, 150 mM NaCl, 1% Triton X-100) plus 1% protease inhibitor cocktail at 4°C for 1 h. The corresponding antibody-conjugated Sepharose beads were added into the lysates supernatant. Following incubation overnight, the beads were washed five times (10 min each). For GST pull-down assay, purified GST-NleL, His-JNKs or cell lysate (appropriate volume) were mixed in total 400 μL reaction system (50 mM Tris-Cl at pH 7.5, 150 mM NaCl, 1% (v/v) Triton X-100, 1 mM EDTA) at 4°C for 6 h. The beads were then pelleted and washed for 5 times (10 min incubation at 4°C for each washing). Then the recovered beads were boiled in 1× SDS-PAGE loading buffer and subjected to SDS-PAGE, followed by Coomassie Blue staining or IB with indicated antibodies.

### Immunofluorescence and microscopy

For immunofluorescence staining, cells were fixed with 4% paraformaldehyde for 20 min at room temperature, permeabilized for 10 min with 0.2% Triton X-100 in PBS, and then blocked for 60 min with 1.0% BSA, followed by incubation with indicated antibodies. All the cell nuclei were counterstained with DAPI before imaging.

### *In vitro* kinase assays

*In vitro* kinase assays were performed as described before [61]. Cells were transfected with plasmids encoding NleL and Flag-JNK1 or JNK2. 24 h after transfection, cells were treated with or without TNFα (10 ng/m1) for 10 min. Cell were then lysed and subjected to IP with anti-Flag M2 beads in M2 buffer (containing 20 mM Tris-HCl, pH 7.6, 250 mM NaCl, 0.5% NP-40, 3.0 mM EDTA, 3.0 mM EGTA). Then the beads with the precipitated JNK proteins were washed and subjected to *in vitro* kinase reaction (30 μl total) containing 1.0 μg of GST-c-Jun (1-79aa) in the kinase buffer (30 mM HEPES, pH 7.4, 3.0 mM DTT, 30 mM PNPP, 0.2 mM NaVO_3_, 30 mM MgCl_2_) supplemented with 2.0 mM ATP. The *in vitro* kinase assays involving human JNKs were carried out at 30°C for 60 min. Reactions were stopped by adding 1 × SDS-PAGE loading buffer and subjected to IB analysis with anti-p-c-Jun antibody.

### Luciferase reporter assay

Luciferase reporter assays were performed as described previously [62]. HEK293T cells seeded in 24-well plates were transiently co-transfected with 50 ng of pAP-1-Luc and 10 ng of pRL-tk-Luc together with or without 500 ng of pCDNA3-NleL, using Lipofectamine 2000 reagent. The total amounts of DNA were kept constant by supplementing empty vector (pCNDA3.0). 24 h after transfection, cells were subjected to TNFα (R&D Systems Inc., 10 ng/ml) or PMA (Sigma, 20 nM) for indicated time. Cells were then lysed in 1 × Passive Lysis Buffer (5 × concentrate diluted in ddH_2_O, Promega) for 15 min at room temperature with vigorous shaking. AP-1 activities were finally determined using the dual luciferase assay kit (Promega). Results were independently replicated for at least three experiments.

### Quantitative RT-PCR (qRT-PCR) analysis

Total RNAs from indicated cells were extracted using TRIzol (Invitrogen). RNA concentrations were determined on Nanodrop ND-1000 sepectrophotometer. 0.1 μg total RNAs were used to cDNA synthesis with the ReverTra Ace qPCR RT kit (FSQ-201, TOYOBO), according to the manufacturer’s instructions. The relative levels of genes expression (normalized to those of *Gapdh*) were assessed in triplicate wells of a 96-well reaction plate by subjecting 10 ng cDNAs per well to a Bio-Rad CFX96 Touch Detection System with SYBR Green chemistry using the following primers:

human *Ccnd1*-forward: 5’-CCGTCCATGCGGAAGATC-3’;

human *Ccnd1*-reverse: 5’ -GAAGACCTCCTCCTCGCACT-3’ [63];

human *Gapdh*-forward: 5’-TGCCCTCAACGACCACTTTG-3’;

human *Gapdh*-reverse: 5’-TTCCTCTTGTGCTCTTGCTGGG-3’ [64].

qRT-PCR data were analyzed on Bio-Rad CFX Manager 3.0.

### Multiple sequence alignments

Protein sequences were retrieved from the RefSeq database, aligned with ClustalW2, and further processed on GeneDoc.

### SEM

Caco-2 cells were fixed in 2.5% glutaraldehyde and then processed for scanning electron microscopy (SEM) analysis as previously described [65]. SEM samples were examined at 25 kV using a FEI Tecnai G2 Spirit TEM (SIBCB, China).

### Yeast two-hybrid assays

Y2H screening with full-length NleL as the bait was performed as described previously [53].

### Statistical analysis

Statistical significance of the data was determined using the Student’s *t*-test. In all experiments, only *P* value of < 0.05 was considered to be statistically significant.

## Supporting information

S1 Fig Characterizing the interaction between NleL and human JNK1 protein. **(A)** The growth of wild-type and Δ*nleL* EHEC O157:H7. Cultures grown to stationary phase were diluted 1:100 into fresh medium, and then the growth of each bacterium was monitored by measuring O.D. 600 at the indicated time points. **(B)** Several candidate genes were identified by using yeast two hybrid (Y2H) system. Full-length *nleL* was cloned into the bait vector pDEST32. Yeast two-hybrid screening was then performed with NleL as the bait, in a human ORFs library. The yeast clones which grow on SD-4 medium (deficient in Leu, Trp, His and Ura) were subjected to sequencing. **(C)** Human JNK1 interacted with NleL *in vitro*. GST-tagged wild type NleL (GST-NleL) or its C753A mutant (GST-NleL-CA) was co-immunoprecipitated with ectopically expressed JNK1. **(D)** GST pull-down assay of full-length NleL or its truncation mutants with JNK1. GST-tagged full-length NleL or its truncation mutants were individually mixed with lysates of the HEK293T cells expressing JNK1. After pull-down, Flag-tagged JNK1 was detected by IB analysis with anti-Flag. Blots are representative of at least three independent experiments.
(TIF)

S2 Fig NleL conjugates mono-ubiquitin or poly-ubiquitin chains to human JNK1 with specific Ub chain linkages. (**A** and **B**) NleL assembled poly-ubiquitin chains *in vitro* or in mammalian cells. *In vitro* ubiquitylation system assembled with purified GST-tagged NleL or C753A mutant, was incubated with E1, E2 (UbcH7), Ub at 37°C for 60 min (**A**). HEK293T cells were co-transfected with His_6_-tagged wild-type NleL or its C753A mutant and HA-tagged Ub. Poly-Ub chains or conjugates were determined by IB analysis with indicated antibodies (**B**). (**C** and **D**) NleL ubiquitylated JNK1 with preferred Ub chain linkages, especially K29-linked Chains. HEK293T cells were transfected with Flag-tagged JNK1 and His_6_-tagged NleL along with wild-type Ub or its mutants. Cell lysates were subjected to immunoprecipitation using anti-Flag M2 beads in denaturing RIPA buffer to enrich Flag-JNK1, followed by IB analysis with indicated antibodies. Blots are representative of at least three independent experiments.
(TIF)

S3 Fig NleL promotes ubiquitylation of JNK1 at multiple sites. **(A)** Several E2s, especially UbcH7, are involved in NleL-mediated JNK1 ubiquitylation *in vivo*. Wild-type or specific-E2 knockdown HEK293T cells were co-transfected with plasmids encoding HA-tagged Ub, Flag-tagged JNK1, His_6_-tagged wild-type NleL. Flag-tagged JNK1 was immunoprecipitated with anti-Flag M2 beads in denaturing RIPA buffer, followed by IB analyses with anti-HA antibody. **(B)** NleL induced ubiquitylation of Flag-JNK1 or its mutants bearing Lys-to-Arg substitution at indicated sites in HEK293T cells. **(C)** The effect of JNK1 K68R mutant on NleL-induced poly-ubiquitylation of JNK1. All the blots are representative of at least three independent experiments.
(TIF)

S4 Fig A sequence alignment of human JNK family proteins. Residues of 100%, over 80% or 60% homology in all aligned sequences are shaded in black, gray, or light gray, respectively. The residues responsible for JNK ubiquitylation by NleL were marked (red). Thr183 and Tyr185, the two conserved phosphorylation sites were labeled (blue color). The isoforms of human JNKs used in the alignment were JNK1α1, JNK2α2 and JNK3α2.
(TIF)

S5 Fig NleL-induced JNK2 ubiquitylation and inactivation. **(A)** NleL promotes ubiquitylation of endogenous JNK2 *in vivo*. Cells expressing HA-tagged Ub and His_6_-tagged NleL (His-NleL) were lysed in 1.0% SDS buffer, boiled at 95°C for 10 min, and then diluted 10 fold in M2 buffer. Then HA-Ub-conjugated protein was enriched with anti-HA antibody and protein G beads, followed by IB analysis with anti-JNK2. **(B)** NleL promotes ubiquitylation of JNK2 with HA-tagged Lys 29 only Ub mutant. Cell lysates were subjected to immunoprecipitation using anti-Flag M2 beads and IB analysis with anti-JNK2 antibody. **(C)** Lys-to-Arg substitutions at Lys 68 and Lys 153 attenuated the ubiquitylation of JNK2. HEK293T cells were transfected with plasmids encoding His_6_-tagged NleL and Flag-tagged JNK2 or the mutant bearing Lys-to-Arg substitution at indicated sites (K68R, K153R or K265R). Cell lysates were subjected to IP with anti-Flag M2 beads in denaturing RIPA buffer, followed by IB analysis with indicated antibodies. **(D)** Wild-type NleL, but not the C753A mutant, inhibits the phosphorylation of ectopically expressed JNK2 in HEK293T cells. Blots are representative of at least three independent experiments.
(TIF)

S6 Fig NleL disturbs the recruitment of JNKs to MKK7. **(A)** NleL impaired the recruitment of endogenous MKK7 to JNK2. Lysates of HEK293T cells expressing Flag-tagged JNK2 together with or without His_6_-tagged NleL were subjected to anti-Flag IP. Then precipitated MKK7 was determined by IB with anti-MKK7. **(B)** NleL attenuated the interaction between ectopically expressed MKK7 and Flag-tagged JNK1 (left) or JNK2 (right). **(C)** NleL had little or no effect on MKK4 phosphorylation. HEK293T cells were stimulated by TNFα (10 ng/ml, 15 min), and then IB analysis was performed with anti-p-MKK4 antibody. Blots are representative of at least three independent experiments.
(TIF)

S7 Fig NleL-mediated ubiquitylation inactivates the JNK/c-Jun signaling pathway. **(A)** NleL abolished TNFα-mediated JNK phosphorylation in the presence of PS-1145, an IKK inhibitor. H1299 cells expressing His_6_-tagged NleL or C753A were treated with TNFα (10 ng/ml) in the presence of PS-1145 (10 μM) for the indicated times. Phosphorylation status of the proteins was determined by IB with respective antibodies. **(B)** Ectopically expressed EGFP-NleL blocked c-Jun activation in HeLa cells with or without TNFα treatment. HeLa cells were stained with anti-p-c-Jun (red) and the nuclears stained with DAPI (blue). Immunofluorescence microscopy was performed. Scale bar, 10 μm. Representative images of at least three independent experiments are shown. The phosphorylation levels of c-Jun in EGFP-positive and EGFP-negative cells were further quantitated (down). Data are represented as the mean ± s.d. from at least four biological replicates, **P < 0.01, ***P < 0.001 (Student’s t-test, n > 4). **(C** and **D)** NleL blocked the transcription activity of AP-1 induced by JNK1 overexpression **(C)** or PMA treatment **(D)**. His-NleL and AP-1 reporter plasmids were transfected to HEK293T cells with (**C**) or without (**D**) Flag-tagged JNK1 for 24 h. Cells were stimulated by PMA (20 nM, 2.5 h) and then subjected to luciferase activity assay. Data are represented as the mean ± s.d., **P < 0.01, ***P < 0.001 (Student’s t-test, n = 3). **(E)** NleL reduced 10% FBS-stimulated protein expression of cyclin D1 in starved cells. HEK293T cells expressing NleL or C753A mutant were serum-starved for 24 h and then stimulated with 10% FBS for indicated times. Then IB blottings were performed to determine protein level of cellular cyclin D1, respectively. **(F)** NleL suppressed thrombin-induced CCND1 expression. Cells expressing NleL or NleL-CA were serum-starved for 24 h, and then subjected to thrombin (1U/mL) treatment for at least 4 h. Blots are representative of at least three independent experiments.
(TIF)

S8 Fig NleL has negligible effect on the ability of *C*. *rodentium* to infect mammalian cells. (**A**) *C*. *rodentium* strain DBS100 (ATCC 51459) was used as wild-type strain. The *nleL*-deleted mutant (Δ*nleL*) and *escR*-deleted mutant (Δ*escR*) had been constructed through a homologous recombination method “Gene doctoring”. Plasmids pTRC99A-NleL and pTRC99A-NleL-C753A are separately introduced to *nleL*-deleted mutant to generated NleL-complemented *nleL*-deleted strain (Δ*nleL* + pNleL) and C753A-complemented *nleL*-deleted strain (Δ*nleL* + pC753A). HeLa cells were infected with indicated *C*. *rodentium* strains with multiplicity of infection (MOI) of 100:1 for 2.5 h, washed with PBS and then re-cultured 2 h in fresh DMEM medium. Infected HeLa cells were thoroughly washed with PBS and then subjected to immunofluorescence microscopy analysis. Shown are representative cell images where anti-LPS staining indicates bacteria (red), DAPI staining marks the nucleus (blue). (**B**) Quantification of relative number of *C*. *rodentium* attached to cells in (A). Bars represent mean ± s.d. from at least five biological replicates, n.s., not significant (Student’s t-test, n>5).
(TIF)

S9 Fig NleL is not required for the ability of *C*. *rodentium* to cause A/E lesions on mammalian cells and colonize to mice colons. (**A**) HeLa cells were infected with indicated *C*. *rodentium* strains with multiplicity of infection (MOI) of 100:1 for 2.5 h, washed with PBS and then re-cultured 2 h in fresh DMEM medium. Infected HeLa cells were thoroughly washed with PBS and then subjected to immunofluorescence microscopy analysis. Shown are representative cell images where anti-LPS staining indicates bacteria (red), DAPI staining marks the nucleus (blue) and F-actin denotes the filamentous actin stained by Cyto-Painter Phalloidin-iFluor 488 Reagent (green). (**B**) 4~5 week-old C57BL/6 mice were intragastrically inoculated with 1 × 10^9^ CFU *C*. *rodentium* strains. Viable stool bacterial counts, measured at 8 days after inoculation, are shown as mean ± s.e.m. of log10 colony-forming units (CFU) per gram faeces.
(TIF)

S10 Fig Chemical inhibition of JNKs with SP600125 promoted EHEC attachment to Caco-2 monolayers by Δ*nleL* strain. **(A)** Quantification of EHEC O157:H7 attached to Caco-2 monolayer (grown for 6 days). Bars represent mean ± s.d. from at least five biological replicates, *P < 0.05, **P < 0.01, ***P < 0.001 (Student’s t-test, n>5). **(B) N**leL enhances the ability of EHEC O157:H7 to attach Caco-2 monolayer (grown for 21 days) by inhibiting JNKs. Caco-2 monolayers (grown for 21 days) treated with DMSO or JNK inhibitor SP600125 (10 μM) were infected with EHEC strains for 2.5 h, then washed with PBS and further cultured for 4 h in fresh medium. After infection, cells were subjected to immunofluorescence microscopy analyses.
(TIF)

S11 Fig Chemical inhibition of JNKs with SP600125 promoted the formation of actin pedestals by Δ*nleL* strain. **(A)** Infection was performed with HeLa cells at a multiplicity of infection of 100:1. Two hours after infection, cells were washed and re-cultured with fresh DMEM for 5 h (replacing medium again at 2.5 h). In the SP600125 group, cells were treated with 5 μM SP600125 for 3 h before infection and 4 ~ 5 h in further culture after infection. Immunofluorescence microscopy was next performed. Representative cell images are shown from three independent experiments. (**B** and **C**) Quantification of actin pedestals (**B**) and EHEC O157:H7 attached to cells (**C**) in (**A**). Bars represent mean ± s.d. from at least five biological replicates, ***P < 0.001, n.s., not significant (Student’s t-test, n>5).
(TIF)
