# Supplementary figures and images for: Bacterial effector NleL promotes enterohemorrhagic E. coli-induced attaching and effacing lesions by ubiquitylating and inactivating JNK
Source: PLoS Pathog. 2017 Jul 28;13(7):e1006534. doi: 10.1371/journal.ppat.1006534 (PMC5549993; doi:10.1371/journal.ppat.1006534)

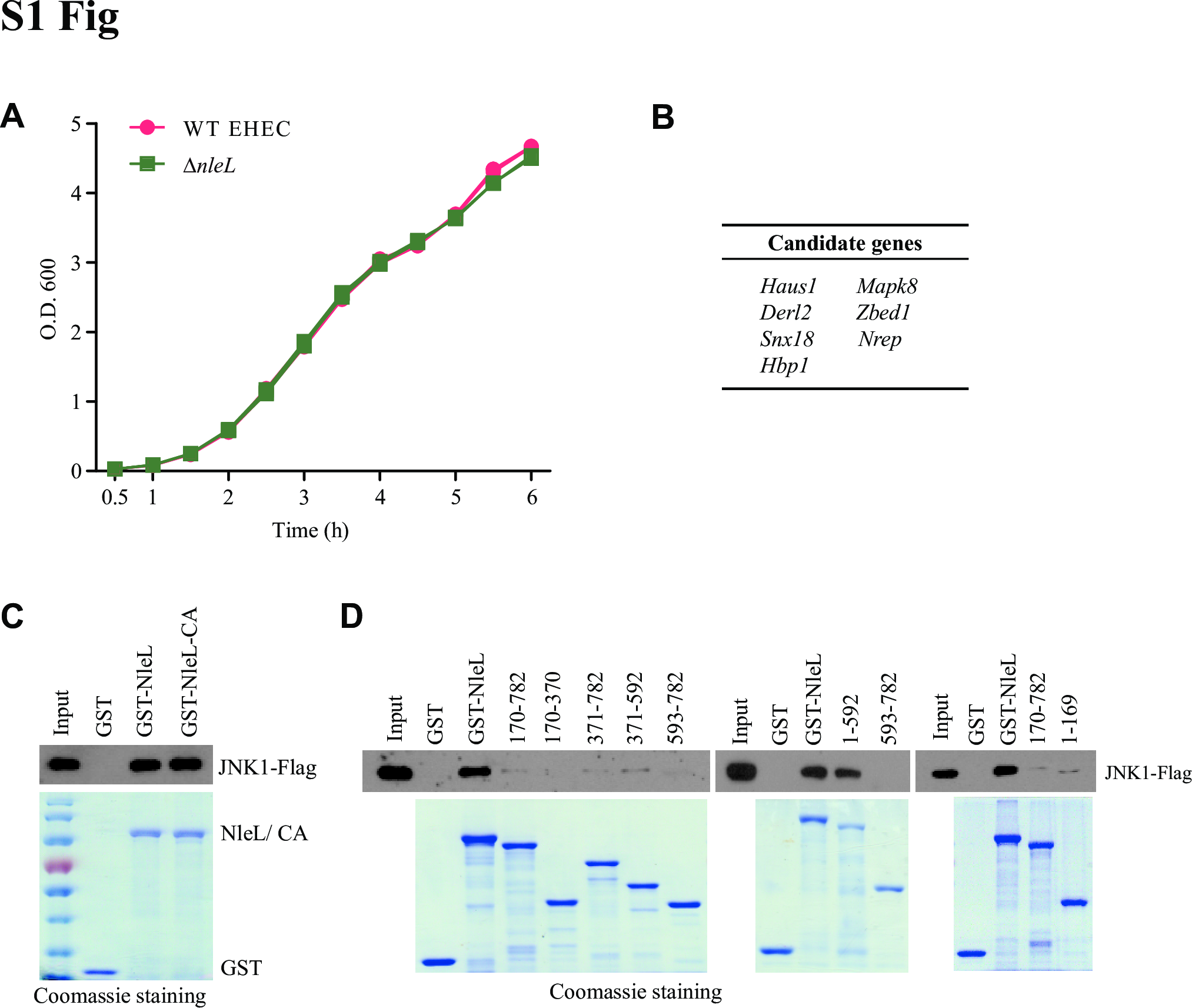

Supplement: S1 Fig — (A) The growth of wild-type and ΔnleL EHEC O157:H7. Cultures grown to stationary phase were diluted 1:100 into fresh medium, and then the growth of each bacterium was monitored by measuring O.D. 600 at the indicated time points. (B) Several candidate genes were identified by using yeast two hybrid (Y2H) system. Full-length nleL was cloned into the bait vector pDEST32. Yeast two-hybrid screening was then performed with NleL as the bait, in a human ORFs library. The yeast clones which grow on SD-4 medium (deficient in Leu, Trp, His and Ura) were subjected to sequencing. (C) Human JNK1 interacted with NleL in vitro. GST-tagged wild type NleL (GST-NleL) or its C753A mutant (GST-NleL-CA) was co-immunoprecipitated with ectopically expressed JNK1. (D) GST pull-down assay of full-length NleL or its truncation mutants with JNK1. GST-tagged full-length NleL or its truncation mutants were individually mixed with lysates of the HEK293T cells expressing JNK1. After pull-down, Flag-tagged JNK1 was detected by IB analysis with anti-Flag. Blots are representative of at least three independent experiments. (TIF) [file ppat.1006534.s001.tif]

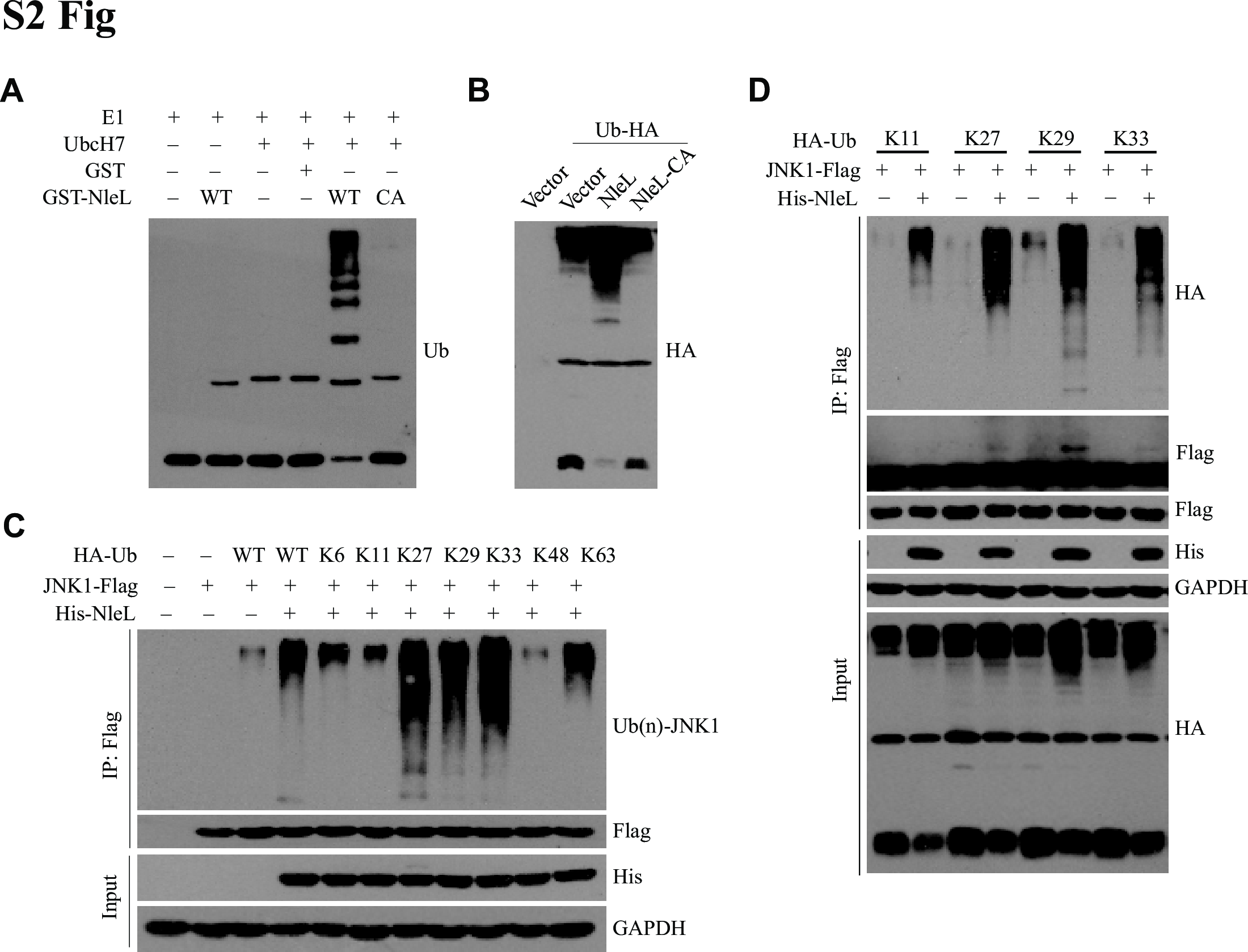

Supplement: S2 Fig — (A and B) NleL assembled poly-ubiquitin chains in vitro or in mammalian cells. In vitro ubiquitylation system assembled with purified GST-tagged NleL or C753A mutant, was incubated with E1, E2 (UbcH7), Ub at 37°C for 60 min (A). HEK293T cells were co-transfected with His6-tagged wild-type NleL or its C753A mutant and HA-tagged Ub. Poly-Ub chains or conjugates were determined by IB analysis with indicated antibodies (B). (C and D) NleL ubiquitylated JNK1 with preferred Ub chain linkages, especially K29-linked Chains. HEK293T cells were transfected with Flag-tagged JNK1 and His6-tagged NleL along with wild-type Ub or its mutants. Cell lysates were subjected to immunoprecipitation using anti-Flag M2 beads in denaturing RIPA buffer to enrich Flag-JNK1, followed by IB analysis with indicated antibodies. Blots are representative of at least three independent experiments. (TIF) [file ppat.1006534.s002.tif]

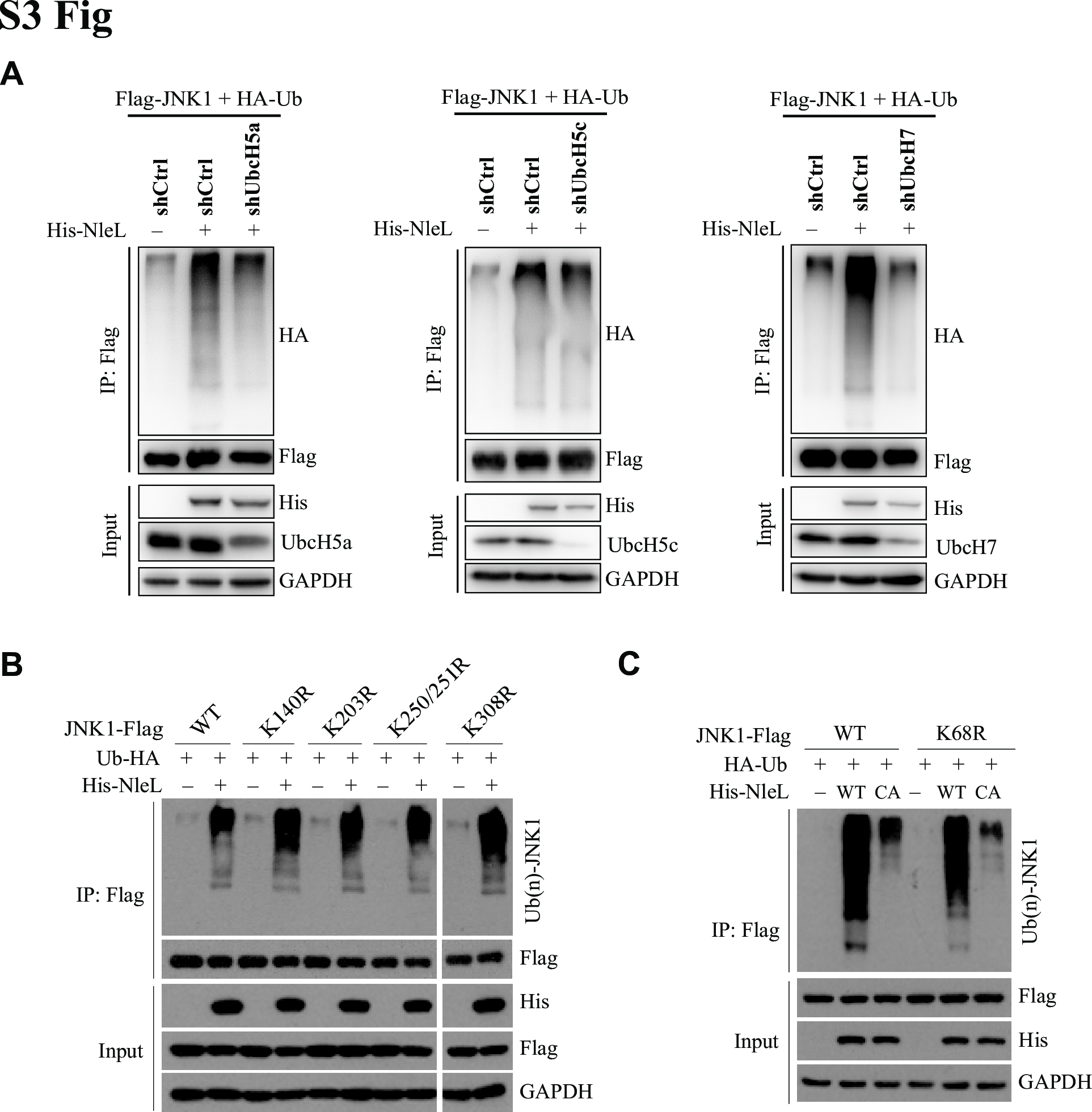

Supplement: S3 Fig — (A) Several E2s, especially UbcH7, are involved in NleL-mediated JNK1 ubiquitylation in vivo. Wild-type or specific-E2 knockdown HEK293T cells were co-transfected with plasmids encoding HA-tagged Ub, Flag-tagged JNK1, His6-tagged wild-type NleL. Flag-tagged JNK1 was immunoprecipitated with anti-Flag M2 beads in denaturing RIPA buffer, followed by IB analyses with anti-HA antibody. (B) NleL induced ubiquitylation of Flag-JNK1 or its mutants bearing Lys-to-Arg substitution at indicated sites in HEK293T cells. (C) The effect of JNK1 K68R mutant on NleL-induced poly-ubiquitylation of JNK1. All the blots are representative of at least three independent experiments. (TIF) [file ppat.1006534.s003.tif]

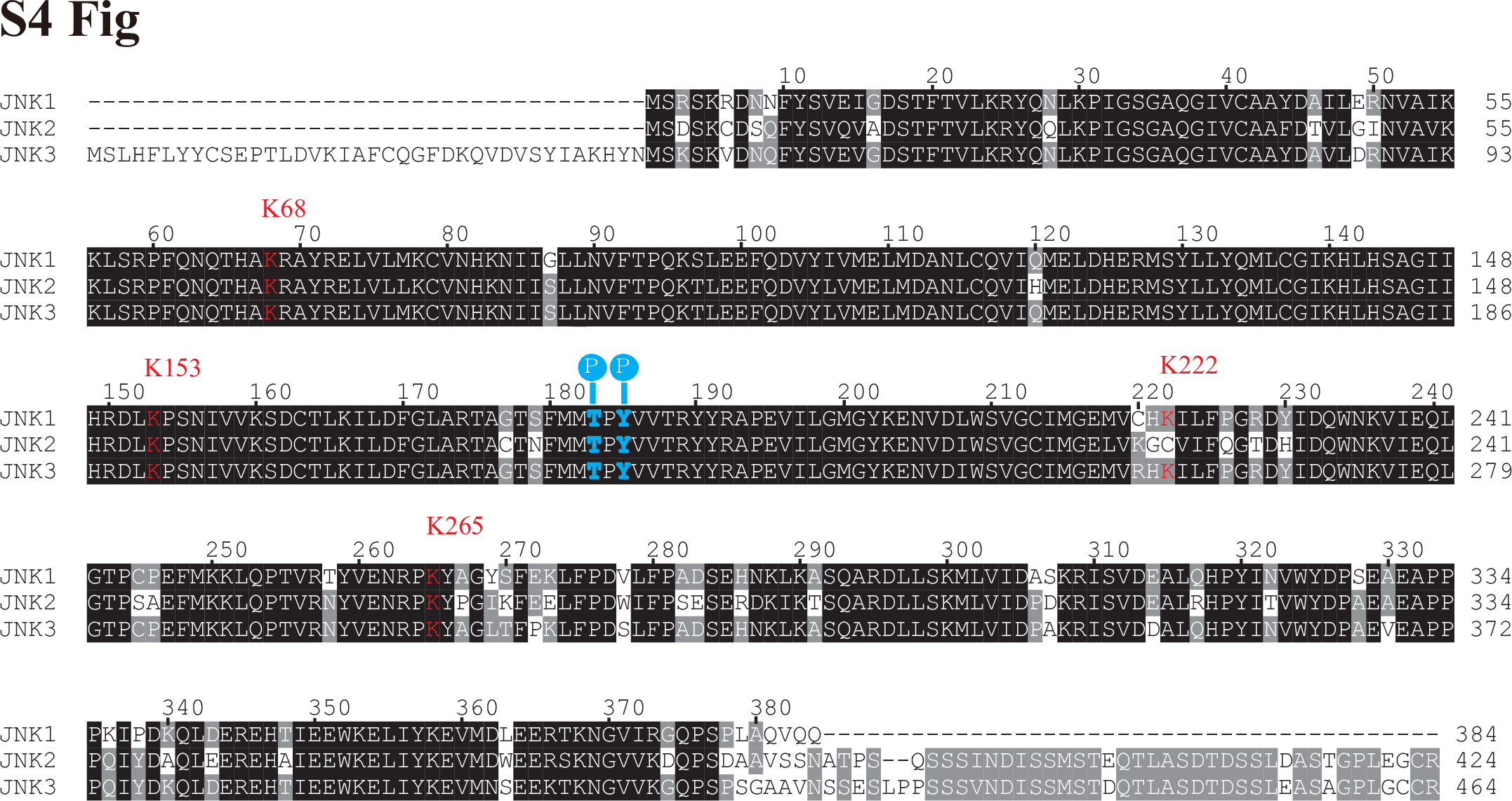

Supplement: S4 Fig — Residues of 100%, over 80% or 60% homology in all aligned sequences are shaded in black, gray, or light gray, respectively. The residues responsible for JNK ubiquitylation by NleL were marked (red). Thr183 and Tyr185, the two conserved phosphorylation sites were labeled (blue color). The isoforms of human JNKs used in the alignment were JNK1α1, JNK2α2 and JNK3α2. (TIF) [file ppat.1006534.s004.tif]

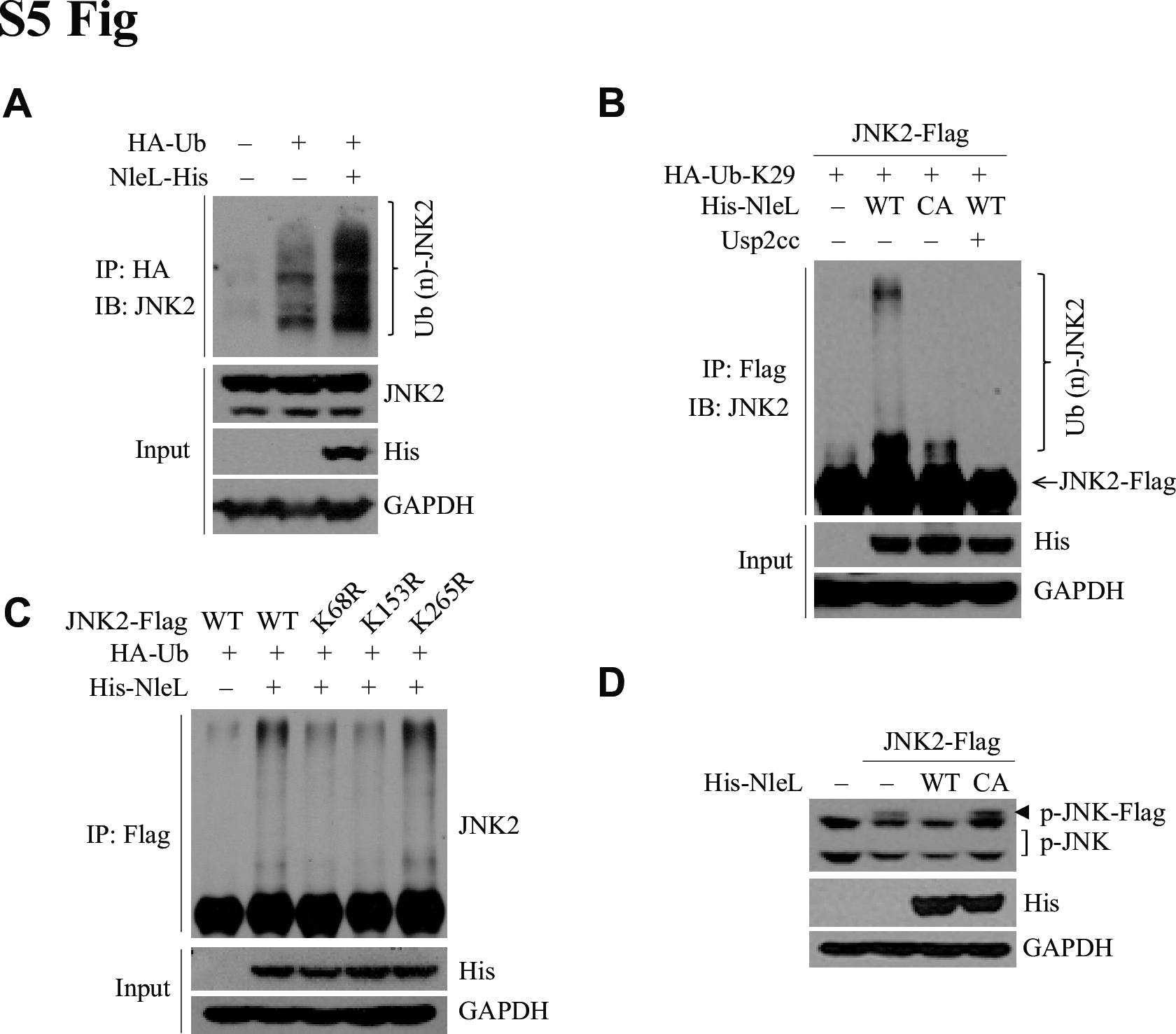

Supplement: S5 Fig — (A) NleL promotes ubiquitylation of endogenous JNK2 in vivo. Cells expressing HA-tagged Ub and His6-tagged NleL (His-NleL) were lysed in 1.0% SDS buffer, boiled at 95°C for 10 min, and then diluted 10 fold in M2 buffer. Then HA-Ub-conjugated protein was enriched with anti-HA antibody and protein G beads, followed by IB analysis with anti-JNK2. (B) NleL promotes ubiquitylation of JNK2 with HA-tagged Lys 29 only Ub mutant. Cell lysates were subjected to immunoprecipitation using anti-Flag M2 beads and IB analysis with anti-JNK2 antibody. (C) Lys-to-Arg substitutions at Lys 68 and Lys 153 attenuated the ubiquitylation of JNK2. HEK293T cells were transfected with plasmids encoding His6-tagged NleL and Flag-tagged JNK2 or the mutant bearing Lys-to-Arg substitution at indicated sites (K68R, K153R or K265R). Cell lysates were subjected to IP with anti-Flag M2 beads in denaturing RIPA buffer, followed by IB analysis with indicated antibodies. (D) Wild-type NleL, but not the C753A mutant, inhibits the phosphorylation of ectopically expressed JNK2 in HEK293T cells. Blots are representative of at least three independent experiments. (TIF) [file ppat.1006534.s005.tif]

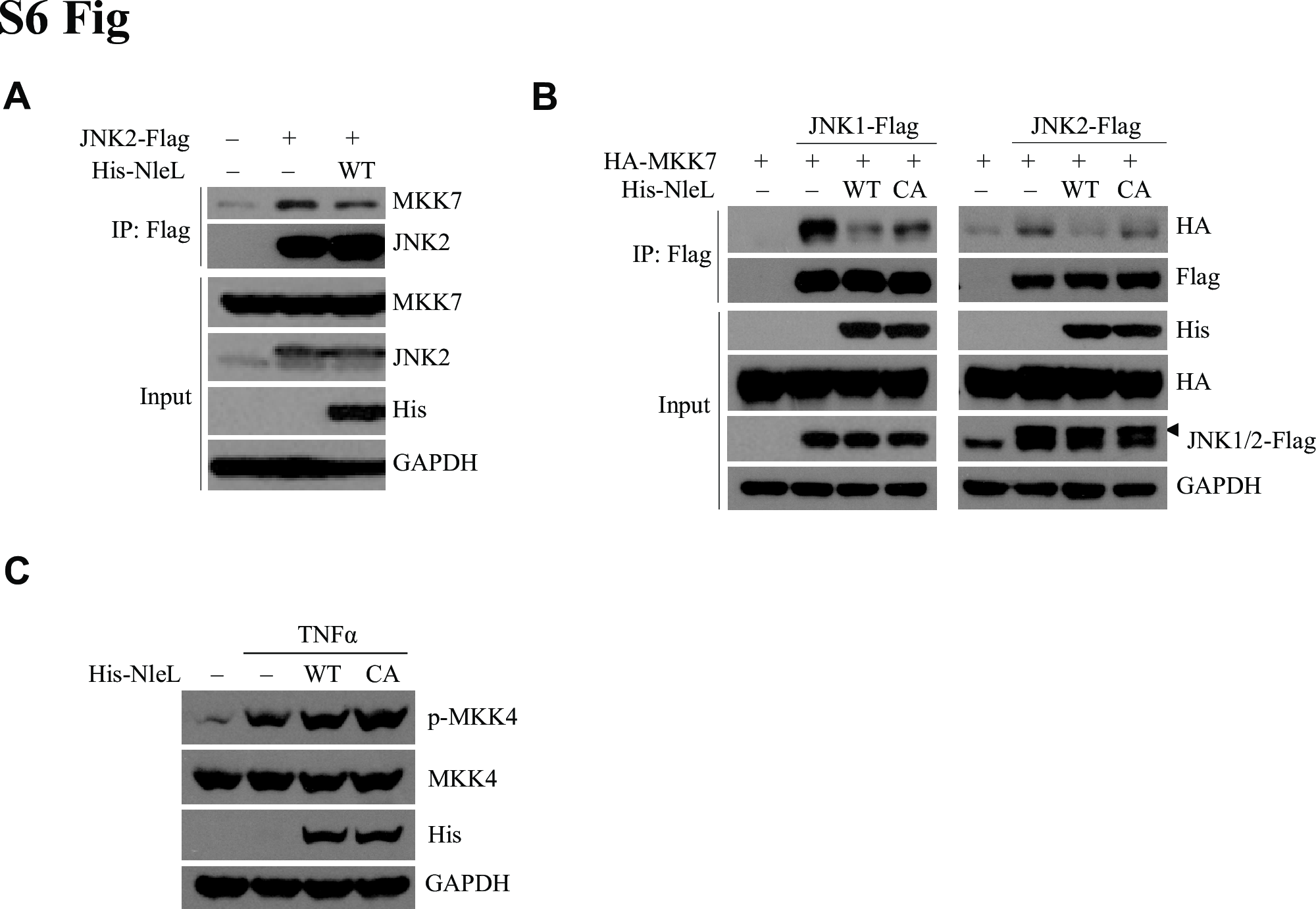

Supplement: S6 Fig — (A) NleL impaired the recruitment of endogenous MKK7 to JNK2. Lysates of HEK293T cells expressing Flag-tagged JNK2 together with or without His6-tagged NleL were subjected to anti-Flag IP. Then precipitated MKK7 was determined by IB with anti-MKK7. (B) NleL attenuated the interaction between ectopically expressed MKK7 and Flag-tagged JNK1 (left) or JNK2 (right). (C) NleL had little or no effect on MKK4 phosphorylation. HEK293T cells were stimulated by TNFα (10 ng/ml, 15 min), and then IB analysis was performed with anti-p-MKK4 antibody. Blots are representative of at least three independent experiments. (TIF) [file ppat.1006534.s006.tif]

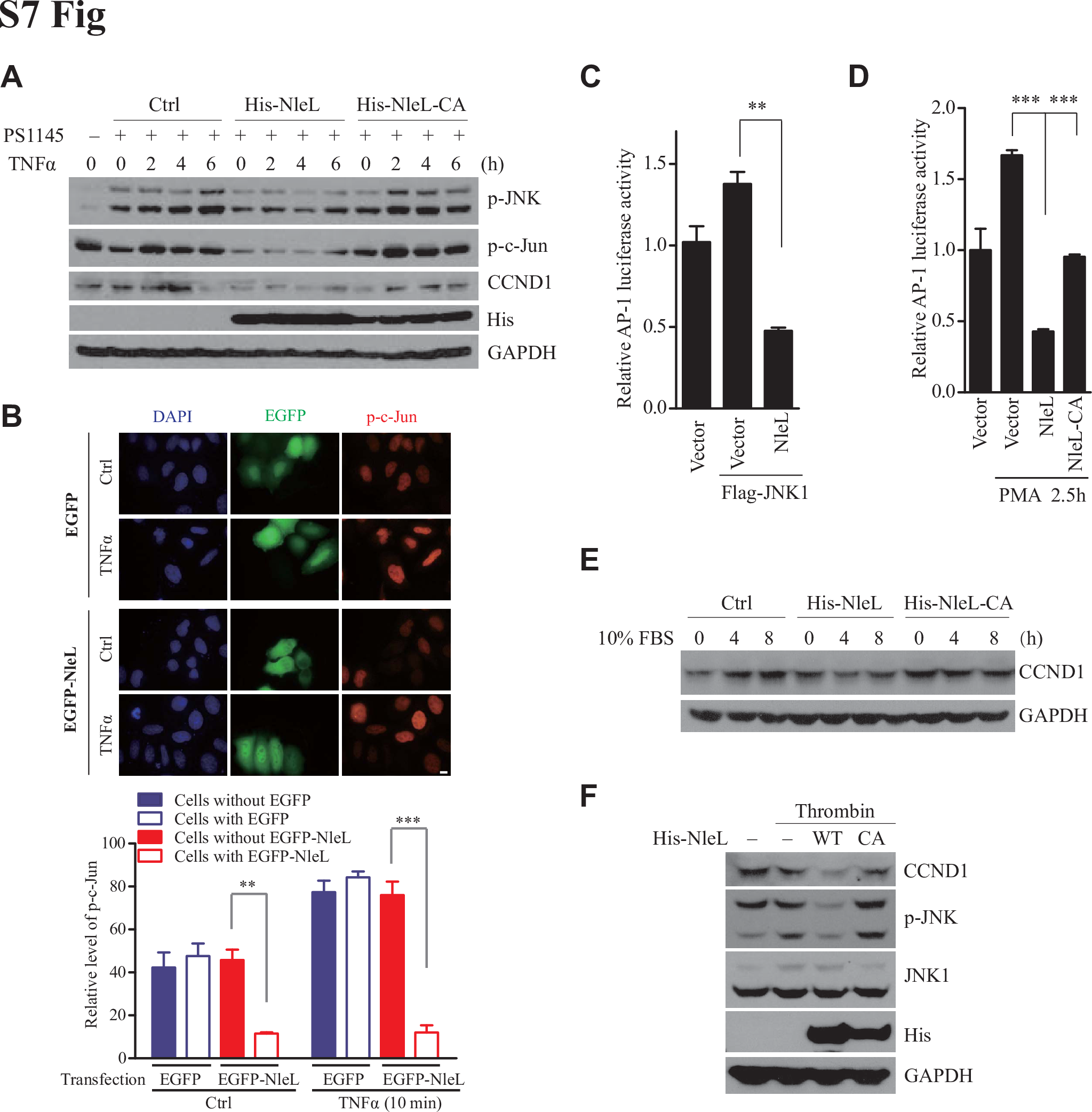

Supplement: S7 Fig — (A) NleL abolished TNFα-mediated JNK phosphorylation in the presence of PS-1145, an IKK inhibitor. H1299 cells expressing His6-tagged NleL or C753A were treated with TNFα (10 ng/ml) in the presence of PS-1145 (10 μM) for the indicated times. Phosphorylation status of the proteins was determined by IB with respective antibodies. (B) Ectopically expressed EGFP-NleL blocked c-Jun activation in HeLa cells with or without TNFα treatment. HeLa cells were stained with anti-p-c-Jun (red) and the nuclears stained with DAPI (blue). Immunofluorescence microscopy was performed. Scale bar, 10 μm. Representative images of at least three independent experiments are shown. The phosphorylation levels of c-Jun in EGFP-positive and EGFP-negative cells were further quantitated (down). Data are represented as the mean ± s.d. from at least four biological replicates, **P < 0.01, ***P < 0.001 (Student’s t-test, n > 4). (C and D) NleL blocked the transcription activity of AP-1 induced by JNK1 overexpression (C) or PMA treatment (D). His-NleL and AP-1 reporter plasmids were transfected to HEK293T cells with (C) or without (D) Flag-tagged JNK1 for 24 h. Cells were stimulated by PMA (20 nM, 2.5 h) and then subjected to luciferase activity assay. Data are represented as the mean ± s.d., **P < 0.01, ***P < 0.001 (Student’s t-test, n = 3). (E) NleL reduced 10% FBS-stimulated protein expression of cyclin D1 in starved cells. HEK293T cells expressing NleL or C753A mutant were serum-starved for 24 h and then stimulated with 10% FBS for indicated times. Then IB blottings were performed to determine protein level of cellular cyclin D1, respectively. (F) NleL suppressed thrombin-induced CCND1 expression. Cells expressing NleL or NleL-CA were serum-starved for 24 h, and then subjected to thrombin (1U/mL) treatment for at least 4 h. Blots are representative of at least three independent experiments. (TIF) [file ppat.1006534.s007.tif]

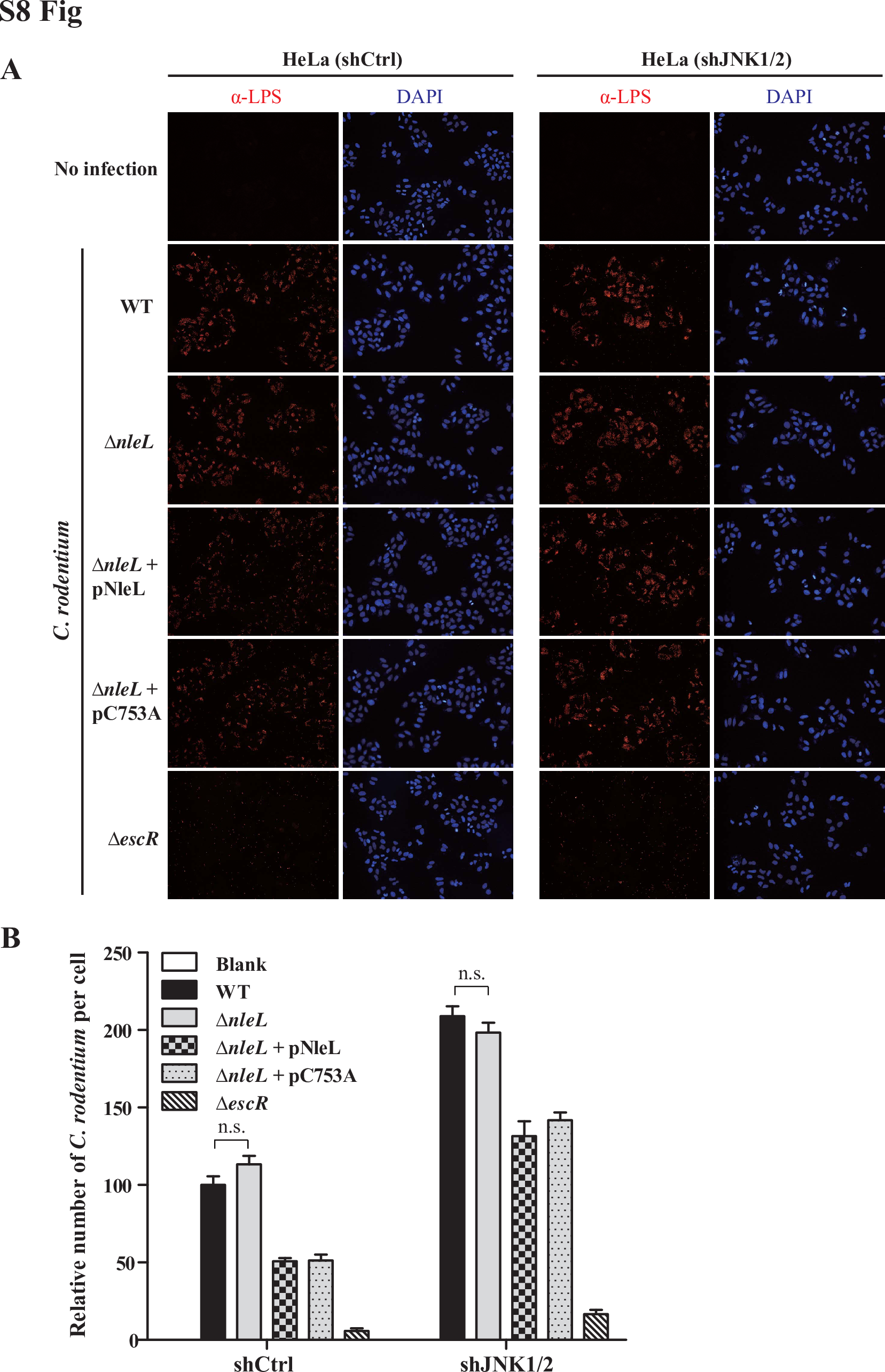

Supplement: S8 Fig — (A) C. rodentium strain DBS100 (ATCC 51459) was used as wild-type strain. The nleL-deleted mutant (ΔnleL) and escR-deleted mutant (ΔescR) had been constructed through a homologous recombination method “Gene doctoring”. Plasmids pTRC99A-NleL and pTRC99A-NleL-C753A are separately introduced to nleL-deleted mutant to generated NleL-complemented nleL-deleted strain (ΔnleL + pNleL) and C753A-complemented nleL-deleted strain (ΔnleL + pC753A). HeLa cells were infected with indicated C. rodentium strains with multiplicity of infection (MOI) of 100:1 for 2.5 h, washed with PBS and then re-cultured 2 h in fresh DMEM medium. Infected HeLa cells were thoroughly washed with PBS and then subjected to immunofluorescence microscopy analysis. Shown are representative cell images where anti-LPS staining indicates bacteria (red), DAPI staining marks the nucleus (blue). (B) Quantification of relative number of C. rodentium attached to cells in (A). Bars represent mean ± s.d. from at least five biological replicates, n.s., not significant (Student’s t-test, n>5). (TIF) [file ppat.1006534.s008.tif]

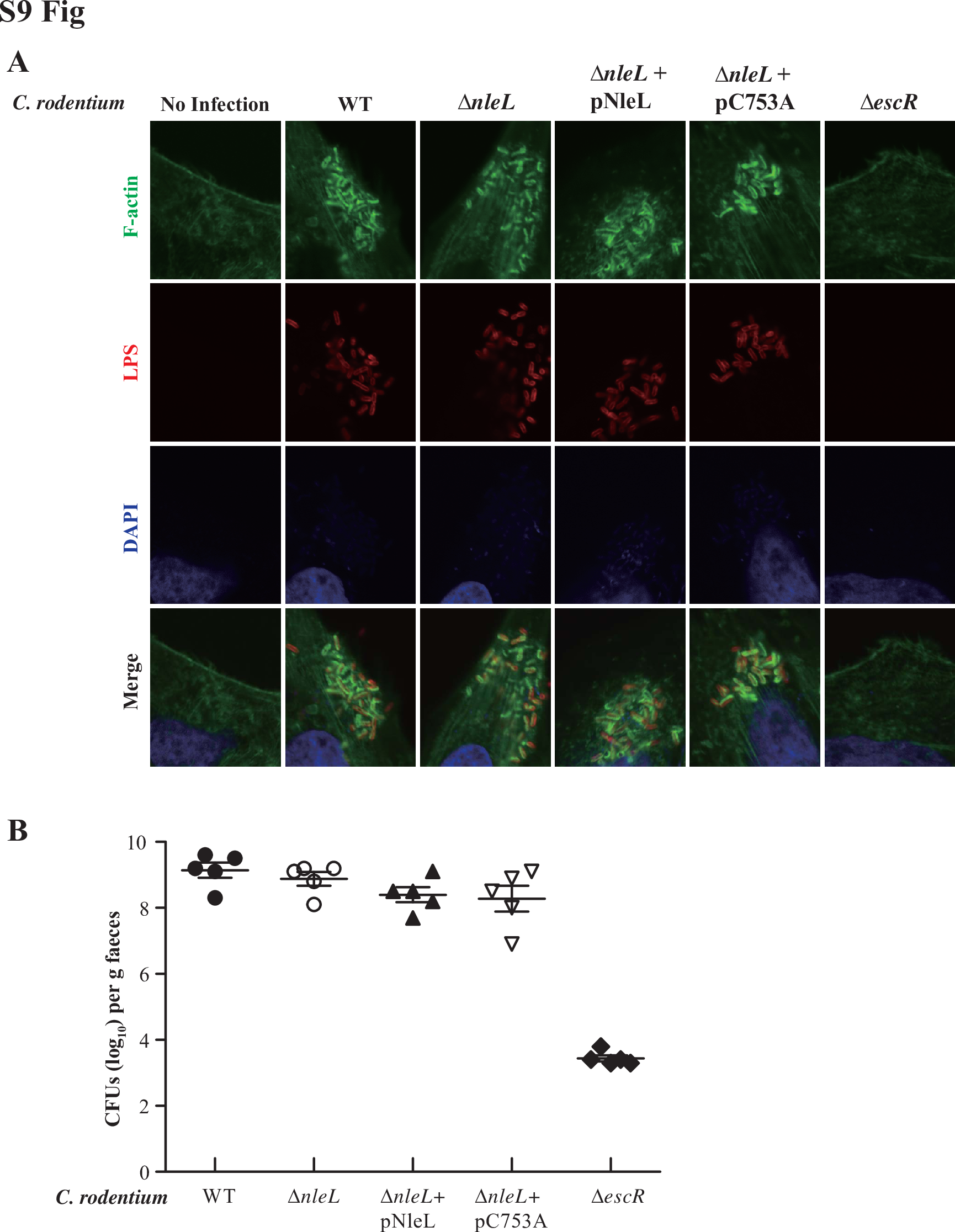

Supplement: S9 Fig — (A) HeLa cells were infected with indicated C. rodentium strains with multiplicity of infection (MOI) of 100:1 for 2.5 h, washed with PBS and then re-cultured 2 h in fresh DMEM medium. Infected HeLa cells were thoroughly washed with PBS and then subjected to immunofluorescence microscopy analysis. Shown are representative cell images where anti-LPS staining indicates bacteria (red), DAPI staining marks the nucleus (blue) and F-actin denotes the filamentous actin stained by Cyto-Painter Phalloidin-iFluor 488 Reagent (green). (B) 4~5 week-old C57BL/6 mice were intragastrically inoculated with 1 × 10^9^ CFU C. rodentium strains. Viable stool bacterial counts, measured at 8 days after inoculation, are shown as mean ± s.e.m. of log10 colony-forming units (CFU) per gram faeces. (TIF) [file ppat.1006534.s009.tif]

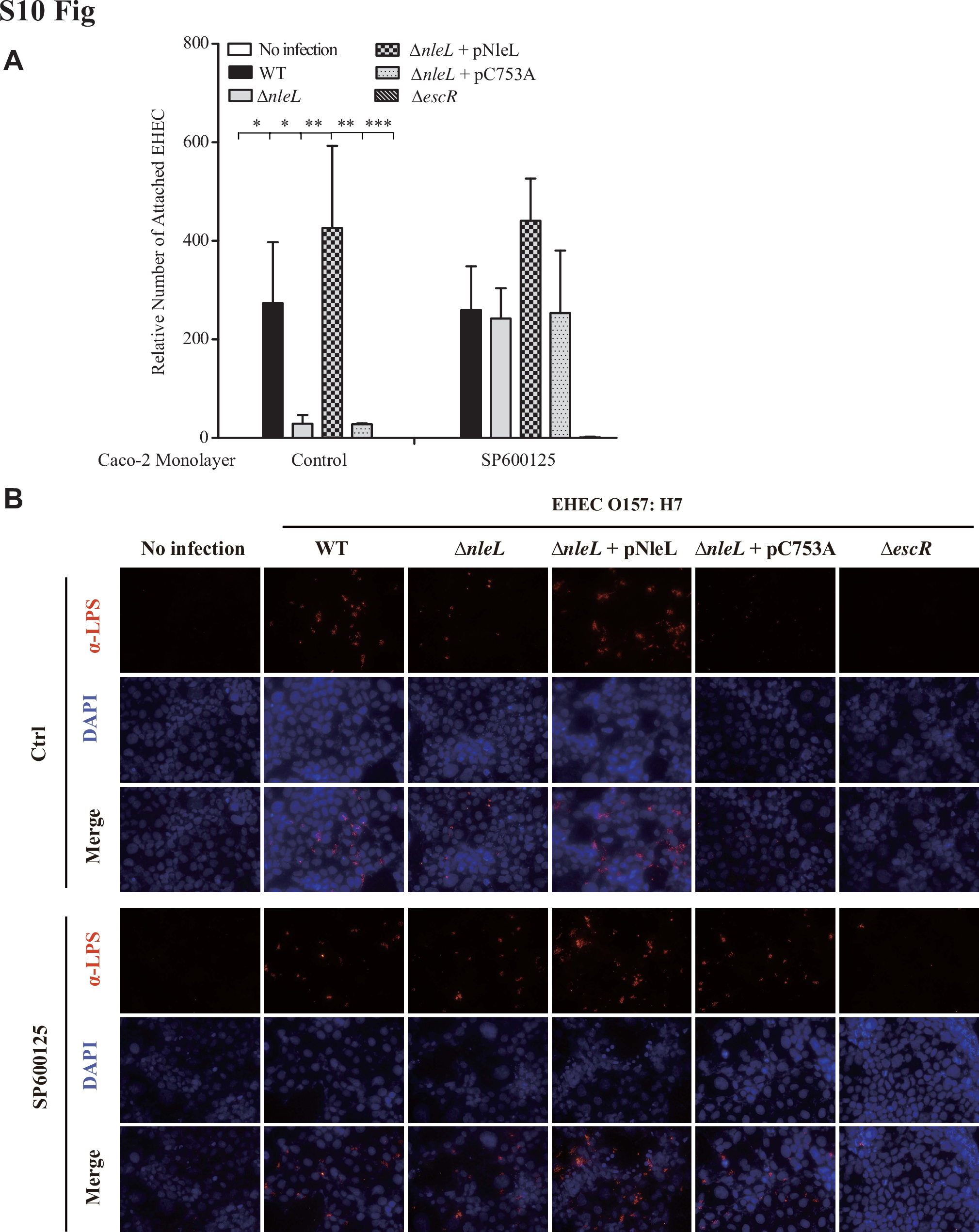

Supplement: S10 Fig — (A) Quantification of EHEC O157:H7 attached to Caco-2 monolayer (grown for 6 days). Bars represent mean ± s.d. from at least five biological replicates, *P < 0.05, **P < 0.01, ***P < 0.001 (Student’s t-test, n>5). (B) NleL enhances the ability of EHEC O157:H7 to attach Caco-2 monolayer (grown for 21 days) by inhibiting JNKs. Caco-2 monolayers (grown for 21 days) treated with DMSO or JNK inhibitor SP600125 (10 μM) were infected with EHEC strains for 2.5 h, then washed with PBS and further cultured for 4 h in fresh medium. After infection, cells were subjected to immunofluorescence microscopy analyses. (TIF) [file ppat.1006534.s010.tif]

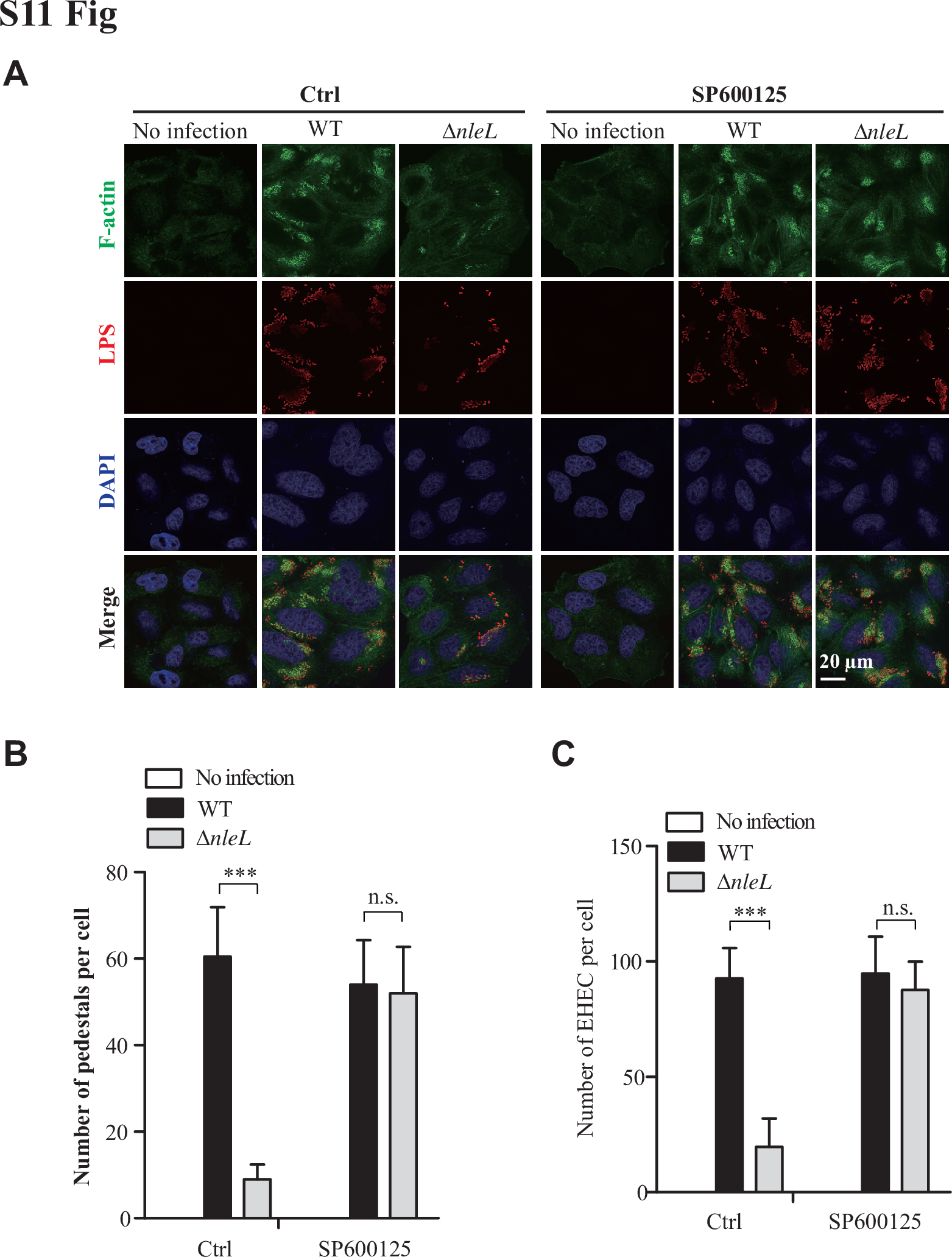

Supplement: S11 Fig — (A) Infection was performed with HeLa cells at a multiplicity of infection of 100:1. Two hours after infection, cells were washed and re-cultured with fresh DMEM for 5 h (replacing medium again at 2.5 h). In the SP600125 group, cells were treated with 5 μM SP600125 for 3 h before infection and 4 ~ 5 h in further culture after infection. Immunofluorescence microscopy was next performed. Representative cell images are shown from three independent experiments. (B and C) Quantification of actin pedestals (B) and EHEC O157:H7 attached to cells (C) in (A). Bars represent mean ± s.d. from at least five biological replicates, ***P < 0.001, n.s., not significant (Student’s t-test, n>5). (TIF) [file ppat.1006534.s011.tif]
